# Identifying and Validating an Acidosis-Related Signature Associated with Prognosis and Tumor Immune Infiltration Characteristics in Pancreatic Carcinoma

**DOI:** 10.1155/2021/3821055

**Published:** 2021-12-28

**Authors:** Pingfei Tang, Weiming Qu, Dajun Wu, Shihua Chen, Minji Liu, Weishun Chen, Qiongjia Ai, Haijuan Tang, Hongbing Zhou

**Affiliations:** Department of Digestive Diseases, Zhuzhou Central Hospital, The Affiliated Zhuzhou Hospital of Xiangya Medical College of Central South University, Zhuzhou, Hunan, China

## Abstract

**Background:**

Acidosis in the tumor microenvironment (TME) is involved in tumor immune dysfunction and tumor progression. We attempted to develop an acidosis-related index (ARI) signature to improve the prognostic prediction of pancreatic carcinoma (PC).

**Methods:**

Differential gene expression analyses of two public datasets (GSE152345 and GSE62452) from the Gene Expression Omnibus database were performed to identify the acidosis-related genes. The Cancer Genome Atlas–pancreatic carcinoma (TCGA-PAAD) cohort in the TCGA database was set as the discovery dataset. Univariate Cox regression and the Kaplan–Meier method were applied to screen for prognostic genes. The least absolute shrinkage and selection operator (LASSO) Cox regression was used to establish the optimal model. The tumor immune infiltrating pattern was characterized by the single-sample gene set enrichment analysis (ssGSEA) method, and the prediction of immunotherapy responsiveness was conducted using the tumor immune dysfunction and exclusion (TIDE) algorithm.

**Results:**

We identified 133 acidosis-related genes, of which 37 were identified as prognostic genes by univariate Cox analysis in combination with the Kaplan–Meier method (*p* values of both methods < 0.05). An acidosis-related signature involving seven genes (*ARNTL2*, *DKK1*, *CEP55*, *CTSV*, *MYEOV*, *DSG2*, and *GBP2*) was developed in TCGA-PAAD and further validated in GSE62452. Patients in the acidosis-related high-risk group consistently showed poorer survival outcomes than those in the low-risk group. The 5-year AUCs (areas under the curve) for survival prediction were 0.738 for TCGA-PAAD and 0.889 for GSE62452, suggesting excellent performance. The low-risk group in TCGA-PAAD showed a higher abundance of CD8+ T cells and activated natural killer cells and was predicted to possess an elevated proportion of immunotherapeutic responders compared with the high-risk counterpart.

**Conclusions:**

We developed a reliable acidosis-related signature that showed excellent performance in prognostic prediction and correlated with tumor immune infiltration, providing a new direction for prognostic evaluation and immunotherapy management in PC.

## 1. Introduction

Pancreatic carcinoma (PC) is one of the most lethal and refractory malignant diseases, with 466,003 new cancer-associated deaths occurring in 2020 worldwide [1]. Pancreatic ductal adenocarcinoma is the major histopathological type, accounting for 85% of cases [2]. Owing to lacking typical signs in the initial stage, approximately 80–85% of patients are diagnosed with unresectable status [3]. The 5-year overall survival probability of PC patients who have undergone surgical operations is only approximately 20% [4]. With the development of genome sequencing technology, the genotype heterogeneity and molecular patterns of PC have been further delineated. The most prevalent mutated genes, such as *KRAS*, *CDKN2A*, *SMAD4*, and *TP53*, participate in the carcinogenesis of pancreatic cancer [5]. Moreover, clinical and preclinical studies are employing advanced targeted therapy based on these altered genes [6]. However, the clinical outcomes of patients with PC are still unclear. A recent real-world study reported that the actual 5-year overall survival rate was lower than 5% when combining all the cases of different stages [7]. Thus, it is imperative to identify novel prognostic indicators to improve the prognostic prediction for PC.

The tumor microenvironment (TME) is composed of various elements including tumor cells, immune and stromal cells, and extracellular components. Due to the unrestricted expansion and abnormal vasculature, the inner parts of the bulk tumor tissues suffer from different levels of insufficient perfusion and hypoxia [8]. As a consequence, with the accumulation of lactic acid and other acid metabolic intermediates, acidosis becomes a common characteristic of solid tumors [9]. Peritumor and tumor invasive regions have been shown to be acidic in mouse tumor models [10]. Acidosis is closely connected with the invasive site at the tumor-stromal interface in human breast cancer tissues [11] and promotes the epithelial-to-mesenchymal transition (EMT) phenotype, affecting the cell viability in several different types of human cancer cells [12, 13]. Another study revealed that the knockdown of acid-sensing ion channels suppressed the EMT of PC cells exposed to acidity and inhibited distant metastasis in a xenograft mouse model [14]. In addition, acidosis contributed to immune cell dysfunction and immune escape in TME and implied a novel therapeutic target [15, 16]. Therefore, we speculate that acidosis in the TME is closely connected with the overall survival outcomes of PC patients. However, there is an absence of acidosis-related prognostic signatures in PC.

In our study, we attempted to identify and validate an acidosis-related index (ARI) signature to predict the clinical outcomes of PC patients. We further investigated the association of the acidosis-related signature and the underlying molecular mechanism, tumor immune infiltration, and immunotherapy response in PC, thereby providing new insights into the immunotherapy of patients with PC.

## 2. Materials and Methods

### 2.1. Data Collection

The sequencing data (FPKM profiles) of patients in The Cancer Genome Atlas (TCGA)–pancreatic carcinoma (PAAD) cohort were downloaded from the TCGA database. We then transformed the gene expression matrix into log_2_ (TPM + 1) values for further investigation. TCGA–PAAD cohort consists of 177 primary PC tumor samples and 4 normal samples, and its corresponding clinical data were publicly obtained from the cBioPortal database [17]. We further excluded patients whose stage information was missing and whose overall survival time was shorter than 1 month. Finally, 168 patients with PC in the TCGA-PAAD project were included in our study and set as the discovery dataset.

The expression profiles (raw count values) of the sequencing dataset GSE152345, including PC cancer cells treated with or without acidosis, were obtained from the GEO database. Another microarray dataset, GSE62452, with 69 pancreatic tumor specimens and 61 normal tissues, was also collected from the GEO database. In particular, 60 pairs of PC tumors and normal samples were selected for further differential gene expression analyses, and 63 PC patients with detailed stage records and survival times no less than 1 month were selected as the validation dataset. All the detailed clinicopathological characteristics of patients in the discovery and validation datasets were listed in Supplemental Table S1 (Table S1). The total flow chart of our study is illustrated in Supplemental Figure S1 (Fig. S1).

### 2.2. Identifying the Acidosis-Related Genes in PC

Differential expression analyses of the sequencing dataset GSE152345 and microarray dataset GSE62452 were conducted by the “Deseq2” package and “limma” package, respectively. We set the criteria of differentially expressed genes (DEGs) as ∣foldchange | >1.5 and adjusted *p* < 0.05. Acidosis-related genes were identified as the overlap of the DEGs in the above two datasets.

### 2.3. Gene Ontology (GO) Functional Enrichment

Functional annotation analyses of the acidosis-related genes in the GO terms were further performed by the “clusterProfiler” package, and adjusted *q* value < 0.05 indicated the statistical significance.

### 2.4. Construction of the Acidosis-Related Prognostic Model

The acidosis-related signature was established using the TCGA-PAAD dataset. Univariate Cox regression in combination with the Kaplan–Meier method was used to select the significantly prognostic acidosis-related genes (*p* < 0.05). Subsequently, LASSO penalty Cox regression was applied to avoid overfitting and develop the optimal signature. The final scoring formula is defined as follows: risk score = ∑_*k*=1_^*n*^exp*k*∗coef*k*, where exp*k* and coef*k* denote the expression value and the LASSO coefficient of each prognostic gene in the signature, respectively.

### 2.5. Evaluating the Prognostic Model

Acidosis-related index (ARI) risk scores of patients with PC in the discovery dataset (TCGA-PAAD) and the independent validation dataset (GSE62452) were estimated using the above formula. On account of the different types of the two datasets, patients in TCGA-PAAD (sequencing dataset) and GSE62452 (microarray dataset) were split into low- and high-risk groups based on their respective median scores. Survival differences were examined by the Kaplan–Meier curves and log-rank test. The prognostic value of the ARI signature was evaluated by time-dependent receiver operating characteristic (ROC) curves.

### 2.6. Gene Set Enrichment Analysis (GSEA)

Hallmark gene sets (“h.all.v7.4.symbols.gmt”) and immunologic signature gene sets (“c7.immunesigdb.v7.4.symbols.gmt”) were downloaded from the MSigDB database [18]. The GSEA algorithm [19], which can determine the differences in predefined gene sets between different phenotypes via genome-wide expression profiles, was used to identify the differentially enriched pathways (nominal *p* value < 0.05 and adjusted *q* value < 0.25) between the acidosis-related risk groups using GSEA software.

### 2.7. Single-Sample GSEA

The well-defined specific gene signatures of 24 immune cells (Table S2) were collected from published literature [20]. We then used the single-sample GSEA (ssGSEA) method [21] to estimate the abundance of these immune cells in each PC tumor sample.

### 2.8. Prediction of Immunotherapy Responsiveness

The tumor immune dysfunction and exclusion (TIDE) algorithm [22], which has been developed to estimate the immunotherapy response based on the genome-wide expression profiles of pretreatment patients, was applied to calculate TIDE scores and predict the immunotherapeutic responsiveness of the patients with PC.

### 2.9. Statistical Analyses

Statistical differences of numerable variables were examined using the Wilcoxon test or Kruskal-Wallis test, while comparisons of category variables were carried out using the chi-square test or Fisher exact test. A standard of *p* value < 0.05 was set as statistical significance except for other specified situations. We made use of the R software to conduct statistical analyses.

## 3. Results

### 3.1. Identifying Acidosis-Related Genes

**Figure 1:**
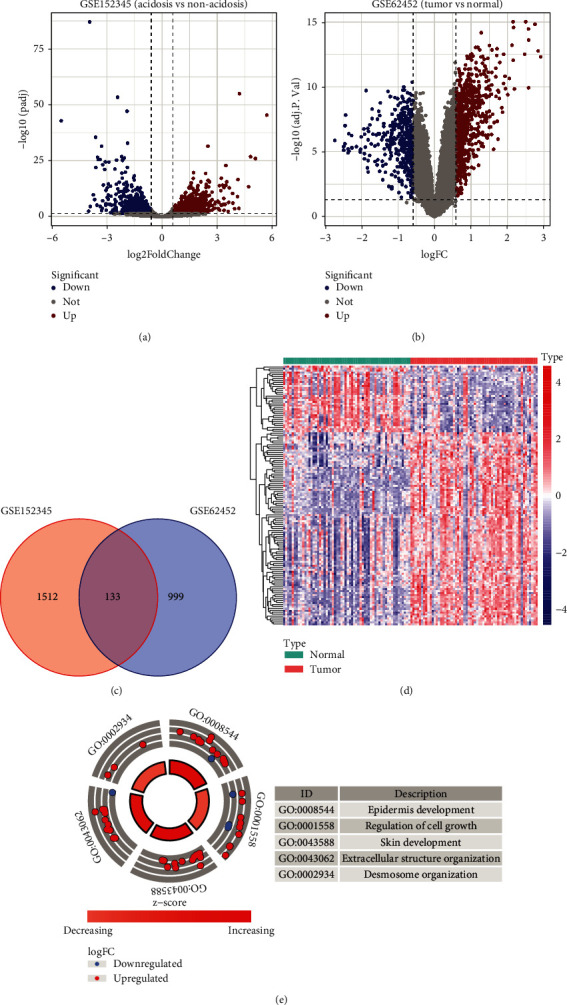
Identification of acidosis-related genes in PC. Volcano plots of DEGs in GSE152345 (a) and GSE62452 (b). (c) Venn plot for the overlapping DEGs between GSE152345 and GSE62452. (d) Heatmap of the 133 acidosis-related genes in 60 pairs of PC tumor and normal tissues in GSE62452. (e) GO functional enrichment analysis of the 133 acidosis-related genes. PC: pancreatic carcinoma; DEGs: differentially expressed genes; GO: Gene Ontology.

A total of 1645 DEGs (807 upregulated and 838 downregulated genes) between acidosis- and nonacidosis-treated PC cells were identified in GSE152345 (Figure 1(a)). Using the same criteria, we acquired 1132 DEGs (720 upregulated and 412 downregulated genes) between 60 pairs of PC tumor tissues and normal samples in GSE62452 (Figure 1(b)). Subsequently, 133 acidosis-related genes in PC were identified by intersecting the above two gene lists (Figure 1(c)). Interestingly, the great majority of these genes showed an increased expression level in tumor specimens compared with normal specimens (Figure 1(d)). Functional annotation analyses revealed that the above acidosis-related genes were significantly enriched in the following GO terms: “epidermis development,” “regulation of cell growth,” “skin development,” “extracellular structure organization,” and “desmosome organization” (Figure 1(e)). This suggests that these acidosis-related genes are closely connected with cell growth and extracellular structure.

### 3.2. Development of Acidosis-Related Signature

**Figure 2:**
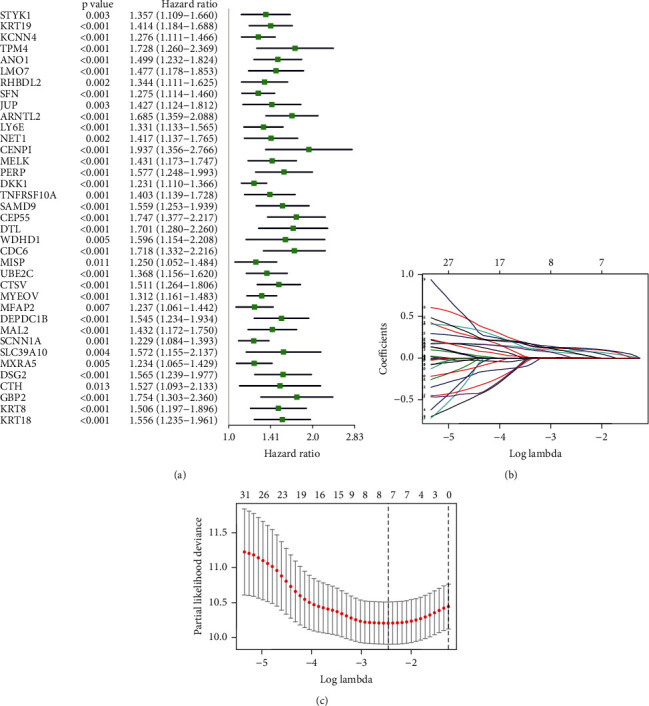
Development of the acidosis-related signature in TCGA-PAAD. (a) Forest plot of 37 prognostic acidosis-related genes identified by univariate Cox regression and Kaplan–Meier method. (b) The tuning parameters of the LASSO penalty Cox regression. (c) Cross-validation of the LASSO regression model, the left vertical dashed line represents the “lambda. min” standard. TCGA: The Cancer Genome Atlas; PAAD: pancreatic carcinoma; LASSO: least absolute shrinkage and selection operator.

The TCGA-PAAD dataset was used as the discovery dataset to develop a prognostic signature. First, 37 out of the above 133 acidosis-related genes were identified as prognostic genes by univariate Cox regression and the Kaplan–Meier method (all *p* < 0.05, Figure 2(a)). Subsequently, LASSO penalty Cox analysis was applied to choose the most contributive variables based on the “lambda. min” standard (Figures 2(b) and 2(c)). An optimal signature involving seven acidosis-related genes (*ARNTL2*, *DKK1*, *CEP55*, *CTSV*, *MYEOV*, *DSG2*, and *GBP2*) was established (Table S3), and the final risk score formula was defined as follows: ARI score = 0.10896∗ARNTL2 expression + 0.00556∗DKK1 expression + 0.12051∗CEP55 expression + 0.09647∗CTSV expression + 0.08222∗MYEOV expression + 0.04550∗DSG2 expression + 0.05175∗GBP2 expression.

### 3.3. Evaluating the Performance of the Acidosis-Related Signature

**Figure 3:**
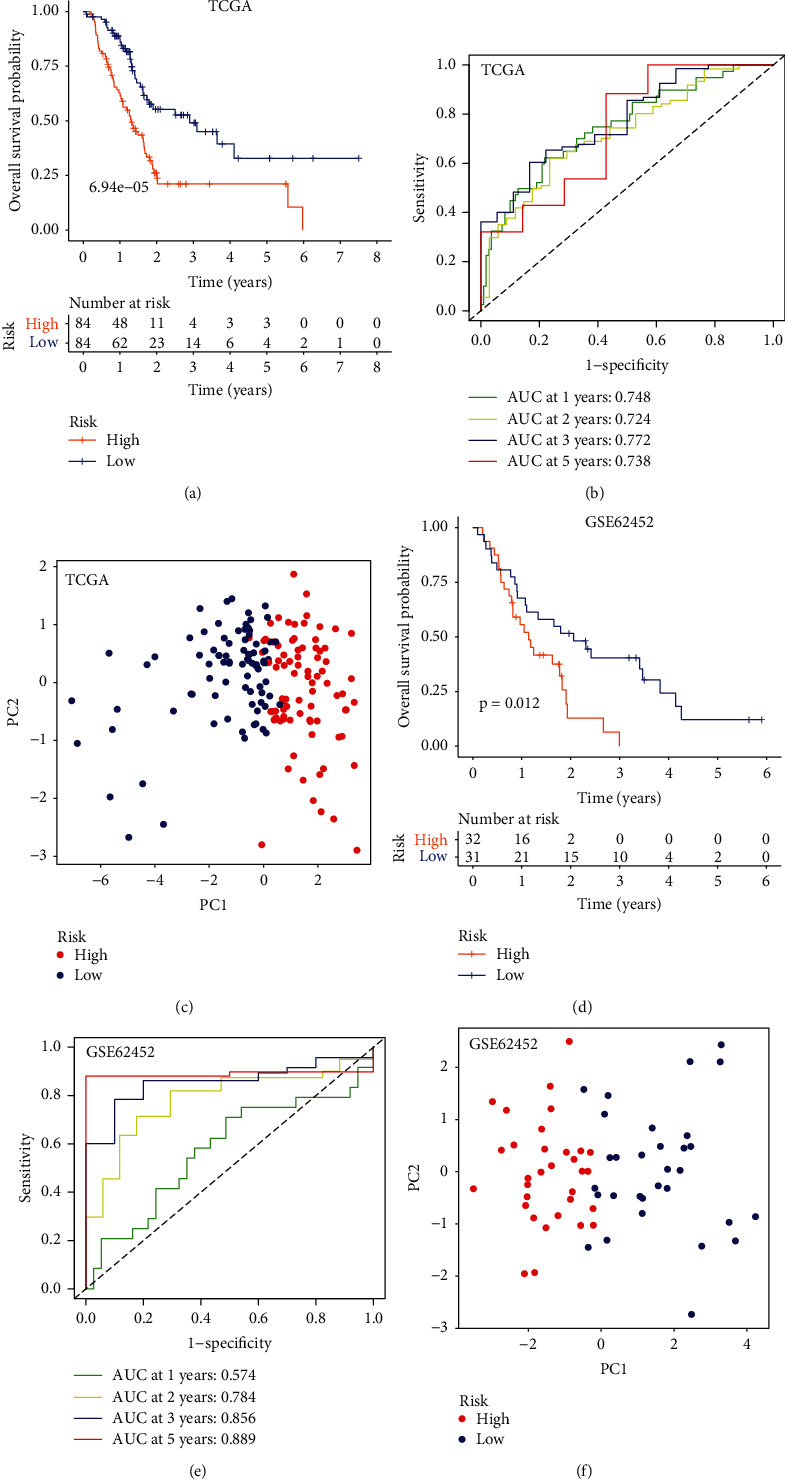
Evaluating the performance of the acidosis-related signature. Kaplan–Meier curves and log-rank test between the high-risk and low-risk groups in TCGA-PAAD (a) and GSE62452 (d). Time-dependent ROC curves of 1-, 2-, 3-, and 5-year survival predictions in TCGA-PAAD (b) and GSE62452 (e). Scatter plots for the PCA results of TCGA-PAAD (c) and GSE62452 (f) according to the acidosis-related signature. ROC: receiver operating characteristic; PCA: principal component analysis.

**Figure 4:**
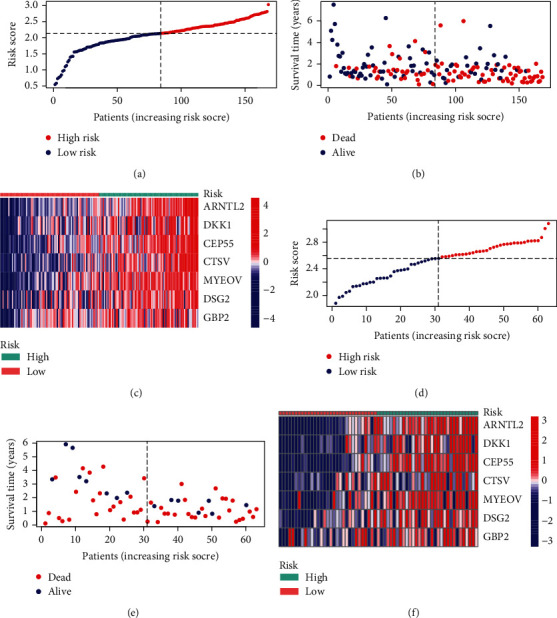
Acidosis-related signature risk scores in the two groups. Distribution of the risk scores and corresponding survival status of patients in TCGA-PAAD (a, b) and GSE62452 (d, e), respectively. Heatmap of the expression levels of the seven key genes within the acidosis-related signature in TCGA-PAAD (c) and GSE62452 (f). PAAD: pancreatic carcinoma.

ARI risk scores for patients with PC in TCGA-PAAD (discovery dataset) and GSE62452 (validation dataset) were calculated using the constructed formula. Patients in each dataset were further assigned to a low- or high-risk group according to their respective median scores (Table S4 for TCGA-PAAD and Table S5 for GSE62452). Notably, patients with high-risk scores showed poorer overall survival outcomes than those with low-risk scores in both TCGA-PAAD (*p* = 6.94*e* − 05, Figure 3(a)) and GSE62452 (*p* = 0.012, Figure 3(d)) datasets. ROC curves showed that AUCs (areas under the curve) of 1-, 2-, 3-, and 5-year survival prediction were 0.748, 0.724, 0.772, and 0.738 in TCGA-PAAD (Figure 3(b)), respectively. The AUCs of 1-, 2-, 3-, and 5-year survival predictions in GSE62452 were 0.574, 0.784, 0.856, and 0.889 (Figure 3(e)), respectively, suggesting an excellent performance for the acidosis-related signature. We further performed the principal component analysis (PCA) for each PC patient in both TCGA-PAAD and GSE62452 according to the expression levels of the seven key genes in the acidosis-related signature. Results showed that there was a distinct expression pattern between the high- and low-risk groups in both TCGA-PAAD (Figure 3(c)) and GSE62452 (Figure 3(f)). Moreover, with the increase of the ARI risk scores in both TCGA-PAAD (Figure 4(a)) and GSE62452 (Figure 4(d)), patients tended to consistently have elevated mortality (Figures 4(b) and 4(e)) and possess relatively elevated expression levels of the seven key genes in the acidosis-related signature (Figures 4(c) and 4(f)), respectively.

### 3.4. Identifying the Independent Prognostic Value of the Acidosis-Related Signature

**Figure 5:**
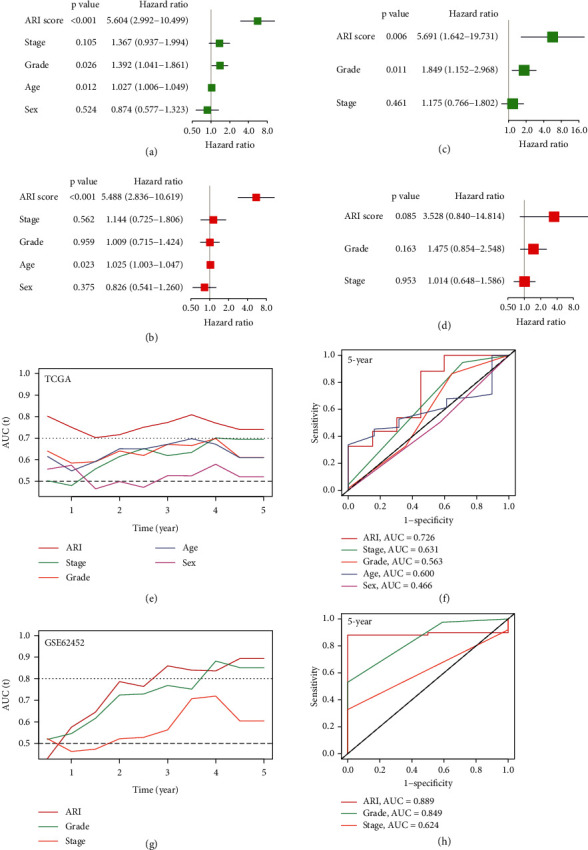
Identifying the acidosis-related signature as an independent prognostic indicator. (a) Univariate Cox analysis and (b) multivariate Cox analysis of critical clinical parameters in TCGA-PAAD. (c) Univariate Cox analysis and (d) multivariate Cox analysis of critical clinical parameters in GSE62452. (e) Time-dependent ROC curves and (f) the AUCs of 5-year survival prediction in TCGA-PAAD. (g) Time-dependent ROC curves and (h) the AUCs of 5-year survival prediction in GSE62452.

Univariate Cox analysis demonstrated that ARI risk scores (HR: 5.604, 95% CI: 2.992−10.499, *p* < 0.001), tumor histopathological grade (HR: 1.392, 95% CI: 1.041−1.861, *p* = 0.026), and age (HR: 1.027, 95% CI: 1.006−1.049, *p* = 0.012) were prognostic factors in patients with PC in TCGA-PAAD (Figure 5(a)). Multivariate Cox analysis further showed that ARI risk scores (HR: 5.488, 95% CI: 2.836−10.619, *p* < 0.001) and age (HR: 1.025, 95% CI: 1.003−1.047, *p* = 0.023) were independent prognostic indicators in TCGA-PAAD (Figure 5(b)). Univariate Cox analysis also indicated that ARI risk scores (HR: 5.691, 95% CI: 1.642−19.731, *p* = 0.006) and tumor histopathological grade (HR: 1.849, 95% CI: 1.152−2.968, *p* = 0.011) were prognostic factors in GSE62452 (Figure 5(c)). Multivariate Cox analysis further showed that the ARI risk score (HR: 3.528, 95% CI: 0.840−14.814, *p* = 0.085) was the most contributive variable for overall survival outcomes compared with other clinical factors in GSE62452 (Figure 5(d)), although the prognostic value did not reach statistical significance (*p* = 0.085). This result might be due to the relatively small sample size and the lack of age records in GSE62452. Time-dependent ROC curves further showed that AUCs of the ARI score for 5-year survival prediction reached 0.726 in TCGA-PAAD (Figures 5(e) and 5(f)) and 0.889 in GSE62452 (Figure 5(g)–5(h)) and were superior to that of other clinical factors such as age, sex, AJCC stage, and tumor grade.

### 3.5. Clinical Correlation Analyses of Acidosis-Related Signature

**Figure 6:**
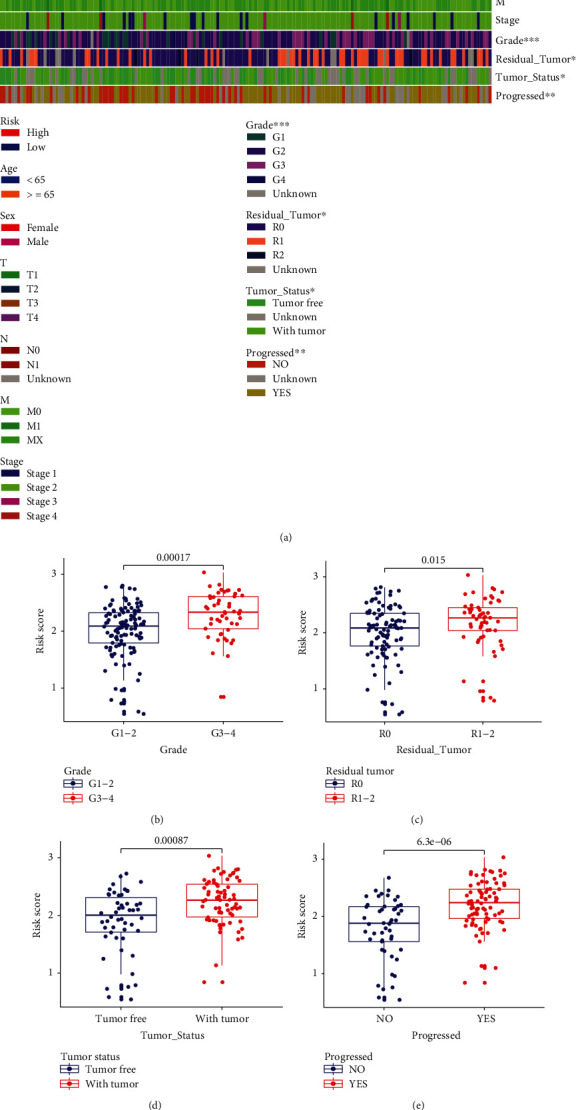
Clinical correlation analyses of the acidosis-related signature in TCGA-PAAD. (a) Heatmap of the distribution landscape of the acidosis-related signature within different clinical characteristics. Comparisons of the acidosis-related index (ARI) risk scores among tumor grade (b), Residual_Tumor (c), Tumor_Status (d), and Progressed (e), respectively. ARI: acidosis-related index.

Due to the remarkable implication in clinical outcomes, we further comprehensively analyzed the correlation between the acidosis-related signature and each clinical factor in the discovery dataset TCGA-PAAD (Figure 6(a)). Interestingly, the acidosis-related high-risk group had a higher proportion of patients with grade 3, “Residual_Tumor (R1),” “Tumor_Status (With Tumor),” and “Progressed (Yes)” than the low-risk group (Fig. S2(A)–S2(D)). Because the ARI risk score was attributed to numerable variables and the above clinical factors were attributed to category variables, we further performed the clinical correlation analyses of the acidosis-related signature by comparing the risk scores between the clinical subgroups via the Wilcoxon test. Patients with grades 3-4, “Residual_Tumor (R1-R2),” “Tumor_Status (With Tumor),” and “Progressed (Yes)” also possessed higher ARI scores than patients with grades 1-2, “Residual_Tumor (R0),” “Tumor_Status (Tumor Free),” and “Progressed (No)”, respectively (Figures 6(b)–6(e)). Furthermore, patients with high ARI scores had adverse disease-free survival outcomes in comparison with those with low ARI scores (*p* = 7.01*e* − 04, Fig. S2(E)). However, there is a lack of correlation between the ARI score and clinical features as sex, age, and stage. A previous study reported that several types of human cancer cells adapted to acidosis exposure could develop into a more mesenchymal-like and invasive phenotype [12]. Interstitial acidification in the tumor microenvironment is associated with the regions of high glycolytic activity and invasiveness [8]. Based on the above evidence, we speculate that our acidosis-related signature may reflect the acidity of tumor tissues and thus is strongly correlated with tumor invasion and progression in patients with PC.

### 3.6. Exploring the Prognostic Value of the Seven Key Acidosis-Related Genes

Differential gene expression analyses showed that the seven key genes in the acidosis-related signature (*ARNTL2*, *DKK1*, *CEP55*, *CTSV*, *MYEOV*, *DSG2*, and *GBP2*) all had higher expression levels in PC tumor specimens than in corresponding normal tissues in GSE62452 (Fig. S3(A)–S3(G)). We further investigated the prognostic value of these seven genes in the TCGA-PAAD dataset. Patients were separated into a high-expression or low-expression group based on the median expression values of *ARNTL2*, *DKK1*, *CEP55*, *CTSV*, *MYEOV*, *DSG2*, and *GBP2*, respectively. Notably, patients with a higher expression level of each of these genes had significantly poorer clinical outcomes than those in the corresponding low-expression group (Fig. S4(A)–S4(G)), suggesting that these seven genes contribute to the progression of PC.

### 3.7. Distinct Molecular Patterns between Acidosis-Related Risk Groups

**Figure 7:**
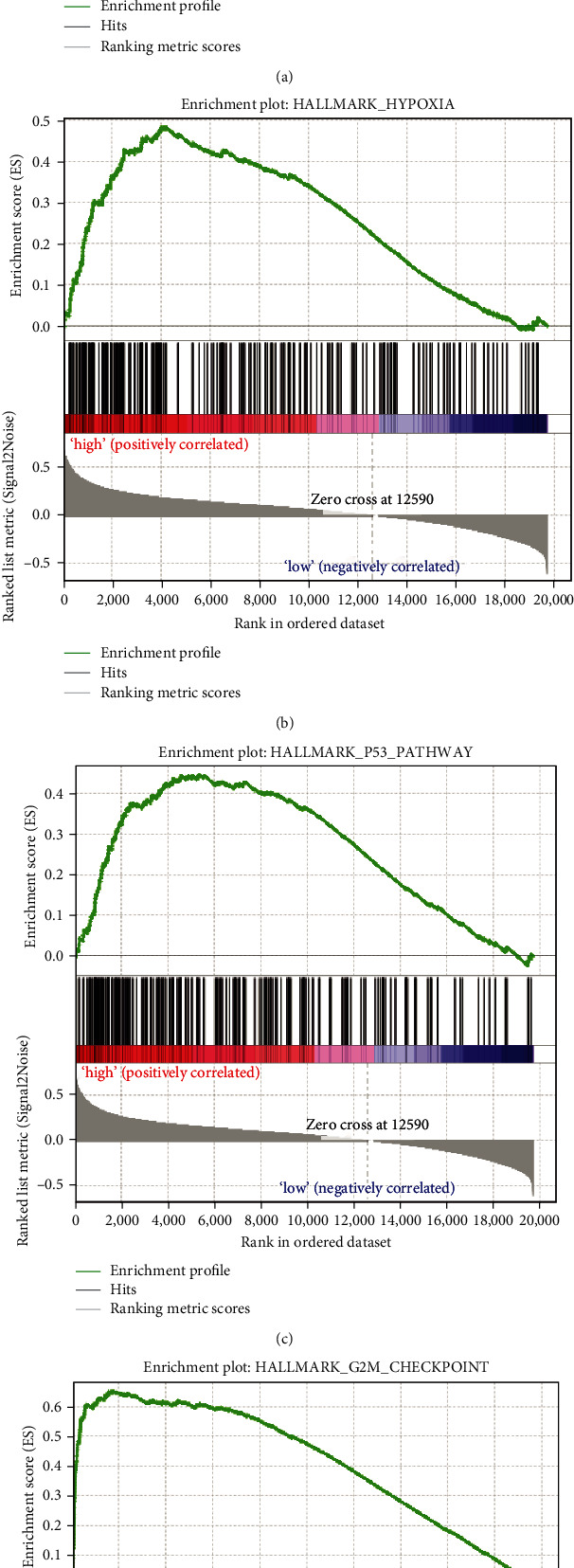
Significantly enriched pathways in the acidosis-related high-risk group in TCGA-PAAD. The enrichment plots of GSEA results in the “HALLMARK_GLYCOLYSIS” (a), “HALLMARK_HYPOXIA” (b), “HALLMARK_P53_PATHWAY” (c), and “HALLMARK_G2M_CHECKPOINT” (d) pathways.

To elucidate the underlying molecular mechanism, the GSEA algorithm was applied to compare the differentially enriched pathways. The acidosis-related high-risk group was significantly enriched in the “GLYCOLYSIS,” “HYPOXIA,” “P53_PATHWAY,” and “G2M_CHECKPOINT” pathways which are tightly linked to tumor aggression and proliferation (Figures 7(a)–7(d)). Acidosis is a common consequence of both glycolysis and hypoxia [9]. On the other side, acidosis in TME can enhance the genome instability such as TP53 mutation and induce the P53 pathway [8, 23]. This most probably explains why these pathways are upregulated in the high ARI score group.

### 3.8. Relationship between Acidosis-Related Signature and Immune Infiltrating Pattern

**Figure 8:**
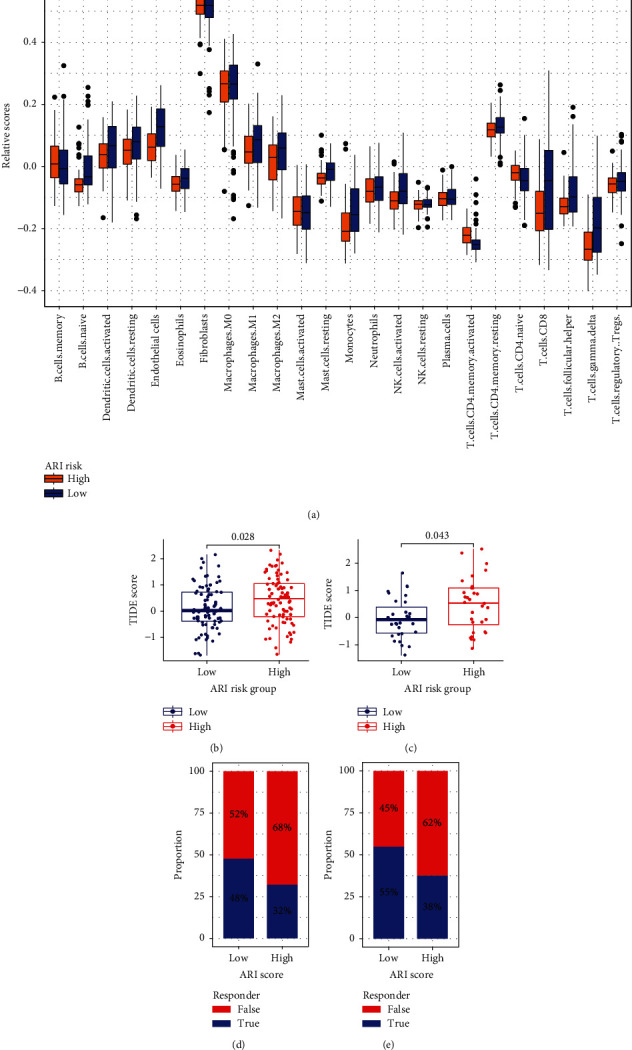
Immune infiltration patterns and the prediction of immunotherapy response in TCGA-PAAD. (a) Comparisons of the infiltrating scores of the 24 immune cells obtained by ssGSEA between the ARI high-risk and low-risk groups in TCGA-PAAD. Comparisons of the TIDE scores (b) and the proportion of the predicted immunotherapeutic responders (d) between the two groups in TCGA-PAAD. Comparisons of the TIDE scores (c) and the proportion of the predicted immunotherapeutic responders (e) between the two groups in GSE62452. ssGSEA: single-sample gene set enrichment analysis; ARI: acidosis-related index; TIDE: tumor immune dysfunction and exclusion.

It is already well known that acidosis at the tumor site can negatively influence T cell function and alter the quality of the immune cells infiltrate [15, 24]. Thus, we further explored the association of the acidosis-related signature and tumor immune infiltrating pattern. GSEA results for the immunologic signature gene sets demonstrated that the acidosis-related high-risk group was enriched in the “NAIVE_VS_24H_IN_VITRO_STIM_CD8_TCELL_DN,” “NAIVE_VS_24H_IN_VITRO_STIM_INFAB_CD8_TCELL_DN,” “NAIVE_VS_72H_IN_VITRO_STIM_CD8_TCELL_DN,” and “NAIVE_VS_72H_IN_VITRO_STIM_IFNAB_CD8_TCELL_DN” pathways (Fig. S5(A)–S5(D)), implying dysfunction of CD8 T cells in the acidosis-related high-risk group. Furthermore, the ssGSEA results of TCGA-PAAD further indicated that the low-risk group possessed a higher abundance of “CD8 T.cells,” “T.cells.gamma.delta,” “NK.cells.activated,” “Dendritic.cells.activated,” “Monocytes,” “Macrophages.M1,” and “Macrophages.M2” than the high-risk group (Figure 8(a)), confirming different immune infiltrating patterns between the acidosis-related risk groups.

### 3.9. Association of Acidosis-Related Signature with Immunotherapy Response

We also assessed the predictive capability of the acidosis-related signature in the immunotherapy response using the TIDE algorithm. Patients in the acidosis-related high-risk group possessed significantly higher TIDE scores than those in the low-risk counterpart for both TCGA-PAAD (*p* = 0.028, Figure 8(b), Table S6) and GSE62452 (*p* = 0.043, Figure 8(c), Table S7) datasets, indicating more tumor immune dysfunction in the acidosis-related high-risk group. Thus, the high-risk group was predicted to possess a relatively lower proportion of immunotherapeutic responders compared with the low-risk group in both TCGA-PAAD (32% versus 48%, Figure 8(d)) and GSE62452 (38% versus 55%, Figure 8(e)) datasets. Pearson's correlation analyses further indicated that the ARI risk scores consistently had a significantly positive correlation with the TIDE scores in both TCGA-PAAD (*r* = 0.2 and *p* = 0.011, Fig. S6(A)) and GSE62452 (*r* = 0.27 and *p* = 0.03, Fig. S6(B)), confirming a close association between the acidosis-related signature and immunotherapy responsiveness. We speculate that the lower responsiveness to immunotherapies in the high-risk group may be due to the poor quality and dysfunction of the tumor-infiltrating lymphocytes in the acidosis condition [15, 16].

## 4. Discussion

PC is one of the most lethal malignant diseases [1] and the actual 5-year overall survival probability is lower than 5% [7]. Thus, it is urgent to develop a novel effective prognostic model in patients with PC. TME is composed of tumor cells, nontumor cells, and diverse extracellular components and contributes substantially to the metastasis process of PC [25, 26]. Hypoxia and acidosis are representative hallmarks of solid tumors and are key regulators of tumor progression [9]. Kandimalla et al. [27] constructed a prognostic signature based on immune, stromal, and proliferation genes to improve the overall survival prediction in patients with PC. Another study also established a hypoxia-related signature associated with decreased abundance of cytotoxic T cells and adverse survival outcomes in PC [28]. However, there is no constructed acidosis-related prognostic signature in patients with PC. Therefore, for the first time, we constructed and validated a reliable acidosis-related signature in two independent PC datasets. Our acidosis-related signature exhibited excellent predictive performance in both TCGA-PAAD and GSE62452 datasets. ROC curves showed that the prognostic signature was superior to other determinants such as age, AJCC stage, and tumor histological grade, suggesting that the acidosis-related signature indeed improves the overall survival prediction in patients with PC.

The acidosis-related signature is composed of seven key genes (*ARNTL2*, *DKK1*, *CEP55*, *CTSV*, *MYEOV*, *DSG2*, and *GBP2*), all of which have elevated expression levels in PC tumor tissues compared with normal tissues and are all related to adverse clinical outcomes in patients with PC. ARNTL2 has been reported to promote the mobility and invasive phenotype of PC cells by mediating the TGF-*β* pathway [29]. The expression level of *DKK1* is increased in the tumor tissues and serum samples of PC patients, and its detection facilitates the diagnosis of early-stage PC [30]. CEP55 enhances the migration and invasion of PC cells via the NF-*κ*B pathway [31]. Studies have also shown that an elevated stromal expression level of *CTSV* [32] or a higher expression level of *MYEOV* [33] is associated with an unfavorable prognosis in PC patients. A previously published study reported that a higher mRNA expression level of *DSG2* predicted a significantly poorer prognosis in TCGA-PAAD, whereas DSG2 protein expression in PC tumor tissues detected by immunohistochemical staining in the in-house cohort showed no significant prognostic value [34]. This contradiction may be due to the different detection methods used in the two PC cohorts. Additionally, a higher protein level of GBP2 was found to be positively correlated with poor clinical outcomes in patients with PC [35]. These results are in line with our conclusion and support that these seven key genes probably act as oncogenes in the progression of PC.

Acidosis in the TME can promote the EMT process and affect cell viability in several types of human cancer cells [12, 13]. Knockdown of acid-sensing ion channels inhibited the distant metastasis of PC cell lines in a xenograft mouse model [14]. In the current study, we also revealed that patients with high acidosis-related scores were predisposed to “Residual_Tumor (R1),” “Tumor_Status (With Tumor),” and “Progressed (Yes)” states, confirming an intensive correlation between the acidosis-related signature and tumor progression in PC patients. Additionally, studies have reported that breast cancer cells exposed to a harsh tumor microenvironment such as hypoxia in combination with acidosis can acquire a more aerobic glycolysis phenotype and possess an enhanced aggressive capability than the controls [36]. The glycolytic activity also modulates the EMT and metastasis cascade in PC [37]. In line with the above evidence, we revealed that the acidosis-related high-risk group was significantly enriched in the “HALLMARK_GLYCOLYSIS” and “HALLMARK_HYPOXIA” pathways, demonstrating that our acidosis-related signature could reflect the acidosis exposure level in PC tumor tissues.

The tumor microenvironment of PC consists of abundant stromal components, and the crosstalk between the numerical stromal cells, immune cells, and cytokines tends to cause immunosuppressive effects [38]. Tumor immune-infiltrating characteristics substantially influence antitumor immunity. The infiltration abundance of T cells varies remarkably in patients with PC [39]. CD8+ cytotoxic T cells are associated with favorable survival outcomes in patients with PC [40, 41]. Natural killer cells play a critical role in the antitumor response of the innate immune system [42]. Enhanced activity of natural killer cells dampens tumor metastasis and is connected with improved recurrence-free survival outcomes in PC patients undergoing surgical resection [43]. Another study revealed that PC patients with a lower proliferated abundance of natural killer cells experienced adverse disease-free and overall survival outcomes [44]. In our study, the acidosis-related high-risk group with an unfavorable prognosis had a lower fraction of CD8 T cells and natural killer cells than the low-risk group, supporting the theory of tumor immunosuppression under acidosis conditions in PC.

Immunotherapy such as immune checkpoint inhibitors has brought promising treatment benefits only in microsatellite instability-high patients with PC but failed to induce a satisfactory response in general PC patients [45]. Thus, there is no “one-fits-all” immunotherapy strategy for PC. Fortunately, tumor extracellular acidosis has been reported to induce immune cell dysfunction and is a novel therapeutic target [15]. Accordingly, we further investigated the relationship between acidosis-related signatures and potential immunotherapy response. Patients in the acidosis-related high-risk group were shown to have higher TIDE scores representing more tumor immune dysfunction and fewer immunotherapeutic responders than those in the low-risk group. Analogously, studies have revealed that exposure to acidosis transforms CD8+ T cells into an anergic state and results in impaired cytolytic activity, whereas neutralizing the acidity to a physiological state can restore the immune function of CD8+ T cells [24]. Neutralization of the tumor extracellular acidity by bicarbonate therapy attenuated the growth of melanoma and PC cell lines and further improved the immunotherapy response in xenografted mouse models [46]. Macrophages also substantially influence the antitumor immunity and the efficacy of cancer immunotherapy [47]. M1 macrophages enhance the tumor phagocytosis, whereas M2 macrophages promote the growth and invasion of tumor cells. However, the exact relationship between macrophage dysfunction and extracellular acidosis in tumor tissues is scarcely reported. Our study showed that both the two subpopulations of macrophages had a relatively elevated level in the low-risk group in comparison with the high-risk group, suggesting the important role of macrophages in tumor immune dysregulation. Additionally, the dendritic cell is the key player that presents the antigen to the cytotoxic T cells and thus is closely involved in the responsiveness to immunotherapy [48]. Activation of dendritic cells strengthens the function of CD8+ T cells and inhibits tumor growth in the PC model [49]. Furthermore, lactic acid produced by tumor tissues can modulate the activity of dendritic cells and impair the differentiation of T cells in a concentration-dependent manner [50]. Analogously, the acidosis-related high-risk group in our study was found to have a decreased fraction of dendritic cells and be less responsive to immunotherapy compared with the low-risk group, confirming the critical role of dendritic cells in antitumor immunity. Collectively, the above evidence suggests that acidity in the tumor microenvironment may serve as a novel target to improve immunotherapy response, and our acidosis-related signature has the potential to discriminate those patients who have less acidosis in TME and might be more responsive to immunotherapy.

However, our study had some limitations. PC tumor specimens in TCGA-PAAD and GSE62452 were collected after surgical resection, and most of these patients were diagnosed with AJCC stage 2 when they were first diagnosed with PC. Owing to the small number of patients with stage 3 or stage 4 disease, our study showed that the AJCC stage was not a significant prognostic factor. Thus, well-designed studies with larger sample sizes and more harmonious properties are needed to further verify the performance of the acidosis-related signature. Furthermore, the seven key genes in the prognostic signature are all risk factors in patients with PC, and their downstream molecular mechanisms need further examination by functional experiments to explore new therapeutic targets. Overall, our acidosis-related signature presents good potential to predict the immunotherapy response, but this notion needs to be tested in rigorously designed clinical trials in the future.

## 5. Conclusions

We developed a reliable acidosis-related signature that showed excellent performance in prognostic prediction and correlated with tumor immune infiltration, providing a new direction for prognostic evaluation and immunotherapy management in PC.

## Data Availability

The expression profiles of GSE152345 and GSE62452 were downloaded from the Gene Expression Omnibus (GEO) database (http://www.ncbi.nlm.nih.gov/geo/), and the expression profile of The Cancer Genome Atlas–pancreatic carcinoma (TCGA-PAAD) project was downloaded from the TCGA database (https://portal.gdc.cancer.gov/).

## Supplementary Materials

Supplementary Materials Figure S1: the flow chart of the present study. Figure S2: comparisons of ARI risk groups between different clinical subgroups in TCGA-PAAD. Comparisons of the distribution differences of the acidosis-related index (ARI) risk groups among tumor grade (a), Residual_Tumor (b), Tumor_Status (c), and Progressed (d), respectively. (e) Kaplan–Meier curves and log-rank test of disease-free survival (DFS) outcomes between ARI high-risk and low-risk groups. ARI: acidosis-related index; DFS: disease-free survival. Figure S3: comparisons of the expression levels of the seven key genes in the acidosis-related signature. Figure S4: overall survival analyses of the seven key genes in the acidosis-related signature in TCGA-PAAD. Figure S5: significantly enriched pathways of immunologic signature gene sets in the acidosis-related high-risk group in TCGA-PAAD. Figure S6: correlation analyses between the ARI risk scores and TIDE scores. Pearson correlation analyses between the ARI risk scores and TIDE scores in TCGA-PAAD (a) and GSE62452 (b). ARI: acidosis-related index; TIDE: tumor immune dysfunction and exclusion. Table S1: clinicopathological characteristics of patients enrolled in the present study. Table S2: the specific gene signatures of 24 immune cells. Table S3: the results of the LASSO Cox regression. Table S4: acidosis-related risk scores of patients in TCGA-PAAD. Table S5: acidosis-related risk scores of patients in GSE62452. Table S6: TIDE scores of patients in TCGA-PAAD. Table S7: TIDE scores of patients in GSE62452.
