# Supplementary material for: Identifying and Validating an Acidosis-Related Signature Associated with Prognosis and Tumor Immune Infiltration Characteristics in Pancreatic Carcinoma
Source: J Immunol Res. 2021 Dec 28;2021:3821055. doi: 10.1155/2021/3821055 (PMC8727107; doi:10.1155/2021/3821055)
Supplement: Supplementary Materials — Figure S1: the flow chart of the present study. Figure S2: comparisons of ARI risk groups between different clinical subgroups in TCGA-PAAD. Comparisons of the distribution differences of the acidosis-related index (ARI) risk groups among tumor grade (a), Residual_Tumor (b), Tumor_Status (c), and Progressed (d), respectively. (e) Kaplan–Meier curves and log-rank test of disease-free survival (DFS) outcomes between ARI high-risk and low-risk groups. ARI: acidosis-related index; DFS: disease-free survival. Figure S3: comparisons of the expression levels of the seven key genes in the acidosis-related signature. Figure S4: overall survival analyses of the seven key genes in the acidosis-related signature in TCGA-PAAD. Figure S5: significantly enriched pathways of immunologic signature gene sets in the acidosis-related high-risk group in TCGA-PAAD. Figure S6: correlation analyses between the ARI risk scores and TIDE scores. Pearson correlation analyses between the ARI risk scores and TIDE scores in TCGA-PAAD (a) and GSE62452 (b). ARI: acidosis-related index; TIDE: tumor immune dysfunction and exclusion. Table S1: clinicopathological characteristics of patients enrolled in the present study. Table S2: the specific gene signatures of 24 immune cells. Table S3: the results of the LASSO Cox regression. Table S4: acidosis-related risk scores of patients in TCGA-PAAD. Table S5: acidosis-related risk scores of patients in GSE62452. Table S6: TIDE scores of patients in TCGA-PAAD. Table S7: TIDE scores of patients in GSE62452. [file 3821055.f1.zip › Supplemental Table S1.docx]

| **Table S1 clinicopathological characteristics** | | |
| --- | --- | --- |
| **Characteristic** | **dataset** | |
|  | **TCGA-PAAD** | **GSE62452** |
| **Total Patients** | 168 | 63 |
| **Age** |  |  |
| <65 years | 76 (45.2%) | NA |
| >=65 years | 92 (54.8%) | NA |
| **Sex** |  |  |
| Female | 77 (45.8%) | NA |
| Male | 91 (54.2%) | NA |
| **Tumor Grade** |  |  |
| grade 1 | 26 (15.5%) | 2 (3.2%) |
| grage 2 | 91 (54.2%) | 31 (49.2%) |
| grade 3 | 47 (28.0%) | 29 (46.0%) |
| grade 4 | 2 (1.2%) | 1 (1.6%) |
| unknown | 2 (1.2%) | 0 |
| **Tumor Stage** |  |  |
| I | 19 (11.3%) | 4 (6.3%) |
| II | 142 (84.5%) | 43 (68.3%) |
| III | 3 (1.8%) | 10 (15.9%) |
| IV | 4 (2.4%) | 6 (9.5%) |
| **Pathologic T Stage** |  |  |
| T1 | 7 (4.2%) | NA |
| T2 | 21 (12.5%) | NA |
| T3 | 137 (81.5%) | NA |
| T4 | 3 (1.8%) | NA |
| **Pathologic N Stage** |  |  |
| N0 | 46 (27.4%) | NA |
| N1 | 118 (70.2%) | NA |
| unknown | 4 (2.4%) | NA |
| **Pathologic M Stage** |  |  |
| M0 | 77 (45.8%) | NA |
| M1 | 4 (2.4%) | NA |
| MX | 87 (51.8%) | NA |
| **Survival Data Type** | OS, DFS | OS |
| OS: overall survival; DFS: disease-free survival | | |
