# Supplementary material for: Identifying and Validating an Acidosis-Related Signature Associated with Prognosis and Tumor Immune Infiltration Characteristics in Pancreatic Carcinoma
Source: J Immunol Res. 2021 Dec 28;2021:3821055. doi: 10.1155/2021/3821055 (PMC8727107; doi:10.1155/2021/3821055)
Supplement: Supplementary Materials — Figure S1: the flow chart of the present study. Figure S2: comparisons of ARI risk groups between different clinical subgroups in TCGA-PAAD. Comparisons of the distribution differences of the acidosis-related index (ARI) risk groups among tumor grade (a), Residual_Tumor (b), Tumor_Status (c), and Progressed (d), respectively. (e) Kaplan–Meier curves and log-rank test of disease-free survival (DFS) outcomes between ARI high-risk and low-risk groups. ARI: acidosis-related index; DFS: disease-free survival. Figure S3: comparisons of the expression levels of the seven key genes in the acidosis-related signature. Figure S4: overall survival analyses of the seven key genes in the acidosis-related signature in TCGA-PAAD. Figure S5: significantly enriched pathways of immunologic signature gene sets in the acidosis-related high-risk group in TCGA-PAAD. Figure S6: correlation analyses between the ARI risk scores and TIDE scores. Pearson correlation analyses between the ARI risk scores and TIDE scores in TCGA-PAAD (a) and GSE62452 (b). ARI: acidosis-related index; TIDE: tumor immune dysfunction and exclusion. Table S1: clinicopathological characteristics of patients enrolled in the present study. Table S2: the specific gene signatures of 24 immune cells. Table S3: the results of the LASSO Cox regression. Table S4: acidosis-related risk scores of patients in TCGA-PAAD. Table S5: acidosis-related risk scores of patients in GSE62452. Table S6: TIDE scores of patients in TCGA-PAAD. Table S7: TIDE scores of patients in GSE62452. [file 3821055.f1.zip › Supplemental Table S2.docx]

| **Table S2 Gene signatures of 24 immune cells** | | |
| --- | --- | --- |
| **Symbol** | **ENSG** | **CellType** |
| ABCB4 | ENSG00000005471 | B.cells.naive |
| ADAM28 | ENSG00000042980 | B.cells.naive |
| BACH2 | ENSG00000112182 | B.cells.naive |
| BCL7A | ENSG00000110987 | B.cells.naive |
| BEND5 | ENSG00000162373 | B.cells.naive |
| BRAF | ENSG00000157764 | B.cells.naive |
| CD22 | ENSG00000012124 | B.cells.naive |
| CD72 | ENSG00000137101 | B.cells.naive |
| CR2 | ENSG00000117322 | B.cells.naive |
| GPR18 | ENSG00000125245 | B.cells.naive |
| HHEX | ENSG00000152804 | B.cells.naive |
| IL4R | ENSG00000077238 | B.cells.naive |
| ZNF263 | ENSG00000006194 | B.cells.naive |
| MEP1A | ENSG00000112818 | B.cells.naive |
| NIPSNAP3B | ENSG00000165028 | B.cells.naive |
| SLC12A1 | ENSG00000074803 | B.cells.naive |
| TCL1A | ENSG00000100721 | B.cells.naive |
| ZNF286A | ENSG00000187607 | B.cells.naive |
| BLK | ENSG00000136573 | B.cells.memory |
| IL7 | ENSG00000104432 | B.cells.memory |
| NPIPB15 | ENSG00000196436 | B.cells.memory |
| SP140 | ENSG00000079263 | B.cells.memory |
| TRAF4 | ENSG00000076604 | B.cells.memory |
| ABCB9 | ENSG00000150967 | Plasma.cells |
| AMPD1 | ENSG00000116748 | Plasma.cells |
| ANGPT4 | ENSG00000101280 | Plasma.cells |
| ATXN8OS | ENSG00000230223 | Plasma.cells |
| C11orf80 | ENSG00000173715 | Plasma.cells |
| CCR10 | ENSG00000184451 | Plasma.cells |
| DENND5B | ENSG00000170456 | Plasma.cells |
| EAF2 | ENSG00000145088 | Plasma.cells |
| GUSBP11 | ENSG00000228315 | Plasma.cells |
| HIST1H2AE | ENSG00000182611 | Plasma.cells |
| HIST1H2BG | ENSG00000187990 | Plasma.cells |
| IGHD | ENSG00000211898 | Plasma.cells |
| IGHE | ENSG00000211891 | Plasma.cells |
| IGLL3P | ENSG00000206066 | Plasma.cells |
| KCNG2 | ENSG00000178342 | Plasma.cells |
| LOC100130100 | Unknown | Plasma.cells |
| MAN1A1 | ENSG00000111885 | Plasma.cells |
| MANEA | ENSG00000172469 | Plasma.cells |
| MAST1 | ENSG00000105613 | Plasma.cells |
| MROH7 | ENSG00000184313 | Plasma.cells |
| MZB1 | ENSG00000170476 | Plasma.cells |
| PAX7 | ENSG00000009709 | Plasma.cells |
| PDK1 | ENSG00000152256 | Plasma.cells |
| RASGRP3 | ENSG00000152689 | Plasma.cells |
| REN | ENSG00000143839 | Plasma.cells |
| SPAG4 | ENSG00000061656 | Plasma.cells |
| ST6GALNAC4 | ENSG00000136840 | Plasma.cells |
| TGM5 | ENSG00000104055 | Plasma.cells |
| TNFRSF17 | ENSG00000048462 | Plasma.cells |
| UGT2B17 | ENSG00000197888 | Plasma.cells |
| CD8A | ENSG00000153563 | T.cells.CD8 |
| CD8B | ENSG00000172116 | T.cells.CD8 |
| CRTAM | ENSG00000109943 | T.cells.CD8 |
| TRAV12-2 | ENSG00000211789 | T.cells.CD8 |
| ANKRD55 | ENSG00000164512 | T.cells.CD4.naive |
| ATHL1 | ENSG00000142102 | T.cells.CD4.naive |
| DSC1 | ENSG00000134765 | T.cells.CD4.naive |
| EPHA1 | ENSG00000146904 | T.cells.CD4.naive |
| FLT3LG | ENSG00000090554 | T.cells.CD4.naive |
| GAL3ST4 | ENSG00000197093 | T.cells.CD4.naive |
| GALR1 | ENSG00000166573 | T.cells.CD4.naive |
| LEF1 | ENSG00000138795 | T.cells.CD4.naive |
| MAP4K2 | ENSG00000168067 | T.cells.CD4.naive |
| UBASH3A | ENSG00000160185 | T.cells.CD4.naive |
| WNT7A | ENSG00000154764 | T.cells.CD4.naive |
| ZNF204P | ENSG00000204789 | T.cells.CD4.naive |
| ZNF324 | ENSG00000083812 | T.cells.CD4.naive |
| EPB41 | ENSG00000159023 | T.cells.CD4.memory.resting |
| ETS1 | ENSG00000134954 | T.cells.CD4.memory.resting |
| FBXL8 | ENSG00000135722 | T.cells.CD4.memory.resting |
| RCAN3 | ENSG00000117602 | T.cells.CD4.memory.resting |
| RPL10L | ENSG00000165496 | T.cells.CD4.memory.resting |
| TRAV13-2 | ENSG00000211791 | T.cells.CD4.memory.resting |
| CDC25A | ENSG00000164045 | T.cells.CD4.memory.activated |
| IFNG | ENSG00000111537 | T.cells.CD4.memory.activated |
| IL17A | ENSG00000112115 | T.cells.CD4.memory.activated |
| IL26 | ENSG00000111536 | T.cells.CD4.memory.activated |
| IL3 | ENSG00000164399 | T.cells.CD4.memory.activated |
| IL4 | ENSG00000113520 | T.cells.CD4.memory.activated |
| IL9 | ENSG00000145839 | T.cells.CD4.memory.activated |
| ORC1 | ENSG00000085840 | T.cells.CD4.memory.activated |
| RRP9 | ENSG00000114767 | T.cells.CD4.memory.activated |
| SKA1 | ENSG00000154839 | T.cells.CD4.memory.activated |
| CHI3L2 | ENSG00000064886 | T.cells.follicular.helper |
| CXCL13 | ENSG00000156234 | T.cells.follicular.helper |
| CXCR5 | ENSG00000160683 | T.cells.follicular.helper |
| FZD3 | ENSG00000104290 | T.cells.follicular.helper |
| ICA1 | ENSG00000003147 | T.cells.follicular.helper |
| ICOS | ENSG00000163600 | T.cells.follicular.helper |
| IL21 | ENSG00000138684 | T.cells.follicular.helper |
| MAP4K1 | ENSG00000104814 | T.cells.follicular.helper |
| PASK | ENSG00000115687 | T.cells.follicular.helper |
| PDCD1 | ENSG00000188389 | T.cells.follicular.helper |
| SLC7A10 | ENSG00000130876 | T.cells.follicular.helper |
| ST8SIA1 | ENSG00000111728 | T.cells.follicular.helper |
| TRIB2 | ENSG00000071575 | T.cells.follicular.helper |
| TSHR | ENSG00000165409 | T.cells.follicular.helper |
| ZAP70 | ENSG00000115085 | T.cells.follicular.helper |
| ZBTB10 | ENSG00000205189 | T.cells.follicular.helper |
| BARX2 | ENSG00000043039 | T.cells.regulatory..Tregs. |
| CD5 | ENSG00000110448 | T.cells.regulatory..Tregs. |
| CD7 | ENSG00000173762 | T.cells.regulatory..Tregs. |
| CD70 | ENSG00000125726 | T.cells.regulatory..Tregs. |
| CEMP1 | ENSG00000205923 | T.cells.regulatory..Tregs. |
| CRISP3 | ENSG00000096006 | T.cells.regulatory..Tregs. |
| CTLA4 | ENSG00000163599 | T.cells.regulatory..Tregs. |
| EFNA5 | ENSG00000184349 | T.cells.regulatory..Tregs. |
| FOXP3 | ENSG00000049768 | T.cells.regulatory..Tregs. |
| FRMD8 | ENSG00000126391 | T.cells.regulatory..Tregs. |
| HIC1 | ENSG00000177374 | T.cells.regulatory..Tregs. |
| HMGB3P30 | ENSG00000213652 | T.cells.regulatory..Tregs. |
| KIRREL | ENSG00000183853 | T.cells.regulatory..Tregs. |
| LAIR2 | ENSG00000167618 | T.cells.regulatory..Tregs. |
| LILRA4 | ENSG00000239961 | T.cells.regulatory..Tregs. |
| LOC126987 | Unknown | T.cells.regulatory..Tregs. |
| NPAS1 | ENSG00000130751 | T.cells.regulatory..Tregs. |
| NTN3 | ENSG00000162068 | T.cells.regulatory..Tregs. |
| PLCH2 | ENSG00000149527 | T.cells.regulatory..Tregs. |
| PMCH | ENSG00000183395 | T.cells.regulatory..Tregs. |
| RYR1 | ENSG00000196218 | T.cells.regulatory..Tregs. |
| SEC31B | ENSG00000075826 | T.cells.regulatory..Tregs. |
| KLHL22 | ENSG00000099910 | T.cells.regulatory..Tregs. |
| SIT1 | ENSG00000137078 | T.cells.regulatory..Tregs. |
| SKAP1 | ENSG00000141293 | T.cells.regulatory..Tregs. |
| SSX1 | ENSG00000126752 | T.cells.regulatory..Tregs. |
| TRAV21 | ENSG00000211801 | T.cells.regulatory..Tregs. |
| TRAV8-6 | ENSG00000211795 | T.cells.regulatory..Tregs. |
| TRAV9-2 | ENSG00000211793 | T.cells.regulatory..Tregs. |
| TYR | ENSG00000077498 | T.cells.regulatory..Tregs. |
| CDH12 | ENSG00000154162 | T.cells.gamma.delta |
| GZMK | ENSG00000113088 | T.cells.gamma.delta |
| TARDBPP1 | ENSG00000233402 | T.cells.gamma.delta |
| TRDC | ENSG00000211829 | T.cells.gamma.delta |
| ZNF442 | ENSG00000198342 | T.cells.gamma.delta |
| CDHR1 | ENSG00000148600 | NK.cells.resting |
| DEFA4 | ENSG00000164821 | NK.cells.resting |
| KLRC3 | ENSG00000205810 | NK.cells.resting |
| KLRF1 | ENSG00000150045 | NK.cells.resting |
| NAALADL1 | ENSG00000168060 | NK.cells.resting |
| S1PR5 | ENSG00000180739 | NK.cells.resting |
| TEP1 | ENSG00000129566 | NK.cells.resting |
| TTC38 | ENSG00000075234 | NK.cells.resting |
| ZNF135 | ENSG00000176293 | NK.cells.resting |
| CCND2 | ENSG00000118971 | NK.cells.activated |
| CDK6 | ENSG00000105810 | NK.cells.activated |
| CTSW | ENSG00000172543 | NK.cells.activated |
| GZMA | ENSG00000145649 | NK.cells.activated |
| IL12RB2 | ENSG00000081985 | NK.cells.activated |
| KIR2DL1 | ENSG00000125498 | NK.cells.activated |
| KIR2DL4 | ENSG00000189013 | NK.cells.activated |
| KIR2DS4 | ENSG00000221957 | NK.cells.activated |
| KIR3DL2 | ENSG00000240403 | NK.cells.activated |
| NCR3 | ENSG00000204475 | NK.cells.activated |
| TNFSF14 | ENSG00000125735 | NK.cells.activated |
| ASGR1 | ENSG00000141505 | Monocytes |
| ASGR2 | ENSG00000161944 | Monocytes |
| CCR2 | ENSG00000121807 | Monocytes |
| CD1D | ENSG00000158473 | Monocytes |
| CD33 | ENSG00000105383 | Monocytes |
| CFP | ENSG00000126759 | Monocytes |
| FCN1 | ENSG00000085265 | Monocytes |
| UPK3A | ENSG00000100373 | Monocytes |
| BHLHE41 | ENSG00000123095 | Macrophages.M0 |
| CHI3L1 | ENSG00000133048 | Macrophages.M0 |
| COL8A2 | ENSG00000171812 | Macrophages.M0 |
| CSF1 | ENSG00000184371 | Macrophages.M0 |
| CXCL5 | ENSG00000163735 | Macrophages.M0 |
| CYP27A1 | ENSG00000135929 | Macrophages.M0 |
| DCSTAMP | ENSG00000164935 | Macrophages.M0 |
| GPC4 | ENSG00000076716 | Macrophages.M0 |
| MARCO | ENSG00000019169 | Macrophages.M0 |
| MMP9 | ENSG00000100985 | Macrophages.M0 |
| PLA2G7 | ENSG00000146070 | Macrophages.M0 |
| PPBP | ENSG00000163736 | Macrophages.M0 |
| ACHE | ENSG00000087085 | Macrophages.M1 |
| ADAMDEC1 | ENSG00000134028 | Macrophages.M1 |
| APOL3 | ENSG00000128284 | Macrophages.M1 |
| APOL6 | ENSG00000221963 | Macrophages.M1 |
| ARRB1 | ENSG00000137486 | Macrophages.M1 |
| CCL19 | ENSG00000172724 | Macrophages.M1 |
| CCL8 | ENSG00000108700 | Macrophages.M1 |
| CD40 | ENSG00000101017 | Macrophages.M1 |
| CXCL10 | ENSG00000169245 | Macrophages.M1 |
| CXCL11 | ENSG00000169248 | Macrophages.M1 |
| CXCL9 | ENSG00000138755 | Macrophages.M1 |
| CYP27B1 | ENSG00000111012 | Macrophages.M1 |
| EBI3 | ENSG00000105246 | Macrophages.M1 |
| HESX1 | ENSG00000163666 | Macrophages.M1 |
| MACF1 | ENSG00000127603 | Macrophages.M1 |
| NOD2 | ENSG00000167207 | Macrophages.M1 |
| PLA1A | ENSG00000144837 | Macrophages.M1 |
| SIGLEC1 | ENSG00000088827 | Macrophages.M1 |
| SLAMF1 | ENSG00000117090 | Macrophages.M1 |
| SLC2A6 | ENSG00000160326 | Macrophages.M1 |
| SOCS1 | ENSG00000185338 | Macrophages.M1 |
| TLR7 | ENSG00000196664 | Macrophages.M1 |
| TNFAIP6 | ENSG00000123610 | Macrophages.M1 |
| TNIP3 | ENSG00000050730 | Macrophages.M1 |
| CCL14 | ENSG00000213494 | Macrophages.M2 |
| CCL18 | ENSG00000006074 | Macrophages.M2 |
| CCL23 | ENSG00000167236 | Macrophages.M2 |
| CD4 | ENSG00000010610 | Macrophages.M2 |
| CD68 | ENSG00000129226 | Macrophages.M2 |
| CLEC4A | ENSG00000111729 | Macrophages.M2 |
| CRYBB1 | ENSG00000100122 | Macrophages.M2 |
| FRMD4A | ENSG00000151474 | Macrophages.M2 |
| HRH1 | ENSG00000196639 | Macrophages.M2 |
| MS4A6A | ENSG00000110077 | Macrophages.M2 |
| NME8 | ENSG00000086288 | Macrophages.M2 |
| NPL | ENSG00000135838 | Macrophages.M2 |
| RENBP | ENSG00000102032 | Macrophages.M2 |
| WNT5B | ENSG00000111186 | Macrophages.M2 |
| ALOX15 | ENSG00000161905 | Dendritic.cells.resting |
| C1orf54 | ENSG00000118292 | Dendritic.cells.resting |
| CD1A | ENSG00000158477 | Dendritic.cells.resting |
| CD1B | ENSG00000158485 | Dendritic.cells.resting |
| CD1C | ENSG00000158481 | Dendritic.cells.resting |
| CD1E | ENSG00000158488 | Dendritic.cells.resting |
| DHRS11 | ENSG00000108272 | Dendritic.cells.resting |
| MMP12 | ENSG00000110347 | Dendritic.cells.resting |
| PPFIBP1 | ENSG00000110841 | Dendritic.cells.resting |
| RNASE6 | ENSG00000169413 | Dendritic.cells.resting |
| SCN9A | ENSG00000169432 | Dendritic.cells.resting |
| TREM2 | ENSG00000095970 | Dendritic.cells.resting |
| ARHGAP22 | ENSG00000128805 | Dendritic.cells.activated |
| BIRC3 | ENSG00000023445 | Dendritic.cells.activated |
| CCL17 | ENSG00000102970 | Dendritic.cells.activated |
| CCL22 | ENSG00000102962 | Dendritic.cells.activated |
| CD86 | ENSG00000114013 | Dendritic.cells.activated |
| CHST7 | ENSG00000147119 | Dendritic.cells.activated |
| CLIC2 | ENSG00000155962 | Dendritic.cells.activated |
| ETV3 | ENSG00000117036 | Dendritic.cells.activated |
| HTR2B | ENSG00000135914 | Dendritic.cells.activated |
| IL12B | ENSG00000113302 | Dendritic.cells.activated |
| MAP3K13 | ENSG00000073803 | Dendritic.cells.activated |
| PDCD1LG2 | ENSG00000197646 | Dendritic.cells.activated |
| ADAMTS3 | ENSG00000156140 | Mast.cells.resting |
| ADRB2 | ENSG00000169252 | Mast.cells.resting |
| FAM124B | ENSG00000124019 | Mast.cells.resting |
| FAM174B | ENSG00000185442 | Mast.cells.resting |
| GFI1 | ENSG00000162676 | Mast.cells.resting |
| HOXA1 | ENSG00000105991 | Mast.cells.resting |
| MS4A2 | ENSG00000149534 | Mast.cells.resting |
| GADD45B | ENSG00000099860 | Mast.cells.resting |
| AZU1 | ENSG00000172232 | Mast.cells.activated |
| CCL1 | ENSG00000108702 | Mast.cells.activated |
| CCL20 | ENSG00000115009 | Mast.cells.activated |
| CXCL3 | ENSG00000163734 | Mast.cells.activated |
| IL1B | ENSG00000125538 | Mast.cells.activated |
| IL5 | ENSG00000113525 | Mast.cells.activated |
| LINC00597 | Unknown | Mast.cells.activated |
| ARVCF | ENSG00000099889 | Mast.cells.activated |
| NOX3 | ENSG00000074771 | Mast.cells.activated |
| NTRK1 | ENSG00000198400 | Mast.cells.activated |
| TEC | ENSG00000135605 | Mast.cells.activated |
| BCL2A1 | ENSG00000140379 | Eosinophils |
| C3AR1 | ENSG00000171860 | Eosinophils |
| CCR3 | ENSG00000183625 | Eosinophils |
| CLC | ENSG00000105205 | Eosinophils |
| DACH1 | ENSG00000165659 | Eosinophils |
| DAPK2 | ENSG00000035664 | Eosinophils |
| DEPDC5 | ENSG00000100150 | Eosinophils |
| EMR1 | ENSG00000174837 | Eosinophils |
| EMR3 | ENSG00000131355 | Eosinophils |
| EPN2 | ENSG00000072134 | Eosinophils |
| GIPR | ENSG00000010310 | Eosinophils |
| GPR183 | ENSG00000169508 | Eosinophils |
| GPR65 | ENSG00000140030 | Eosinophils |
| GPR97 | ENSG00000182885 | Eosinophils |
| IL5RA | ENSG00000091181 | Eosinophils |
| LRMP | ENSG00000118308 | Eosinophils |
| NR4A3 | ENSG00000119508 | Eosinophils |
| OSM | ENSG00000099985 | Eosinophils |
| P2RY10 | ENSG00000078589 | Eosinophils |
| P2RY14 | ENSG00000174944 | Eosinophils |
| P2RY2 | ENSG00000175591 | Eosinophils |
| PDE6C | ENSG00000095464 | Eosinophils |
| PKD2L2 | ENSG00000078795 | Eosinophils |
| RGS1 | ENSG00000090104 | Eosinophils |
| RNASE2 | ENSG00000169385 | Eosinophils |
| RRP12 | ENSG00000052749 | Eosinophils |
| SAMSN1 | ENSG00000155307 | Eosinophils |
| SMPD3 | ENSG00000103056 | Eosinophils |
| SMPDL3B | ENSG00000130768 | Eosinophils |
| TRPM6 | ENSG00000119121 | Eosinophils |
| ZNF165 | ENSG00000197279 | Eosinophils |
| ZNF222 | ENSG00000159885 | Eosinophils |
| AQP9 | ENSG00000103569 | Neutrophils |
| BTNL8 | ENSG00000113303 | Neutrophils |
| C5AR1 | ENSG00000197405 | Neutrophils |
| CDA | ENSG00000158825 | Neutrophils |
| CEACAM3 | ENSG00000170956 | Neutrophils |
| CREB5 | ENSG00000146592 | Neutrophils |
| CSF3R | ENSG00000119535 | Neutrophils |
| CXCR1 | ENSG00000163464 | Neutrophils |
| CXCR2 | ENSG00000180871 | Neutrophils |
| FAM212B | ENSG00000197852 | Neutrophils |
| FAM65B | ENSG00000111913 | Neutrophils |
| FCGR3B | ENSG00000162747 | Neutrophils |
| FFAR2 | ENSG00000126262 | Neutrophils |
| FPR1 | ENSG00000171051 | Neutrophils |
| FPR2 | ENSG00000171049 | Neutrophils |
| HAL | ENSG00000084110 | Neutrophils |
| HSPA6 | ENSG00000173110 | Neutrophils |
| LST1 | ENSG00000204482 | Neutrophils |
| MAK | ENSG00000111837 | Neutrophils |
| MEFV | ENSG00000103313 | Neutrophils |
| MGAM | ENSG00000257335 | Neutrophils |
| MMP25 | ENSG00000008516 | Neutrophils |
| MNDA | ENSG00000163563 | Neutrophils |
| MXD1 | ENSG00000059728 | Neutrophils |
| NFE2 | ENSG00000123405 | Neutrophils |
| P2RY13 | ENSG00000181631 | Neutrophils |
| PGLYRP1 | ENSG00000008438 | Neutrophils |
| REPS2 | ENSG00000169891 | Neutrophils |
| STEAP4 | ENSG00000127954 | Neutrophils |
| TNFRSF10C | ENSG00000173535 | Neutrophils |
| TREM1 | ENSG00000124731 | Neutrophils |
| TREML2 | ENSG00000112195 | Neutrophils |
| VNN2 | ENSG00000112303 | Neutrophils |
| VNN3 | ENSG00000093134 | Neutrophils |
| ACVRL1 | ENSG00000139567 | Endothelial cells |
| APLN | ENSG00000171388 | Endothelial cells |
| BCL6B | ENSG00000161940 | Endothelial cells |
| BMP6 | ENSG00000153162 | Endothelial cells |
| BMX | ENSG00000102010 | Endothelial cells |
| CDH5 | ENSG00000179776 | Endothelial cells |
| CLEC14A | ENSG00000176435 | Endothelial cells |
| CXorf36 | ENSG00000147113 | Endothelial cells |
| EDN1 | ENSG00000078401 | Endothelial cells |
| ELTD1 | ENSG00000162618 | Endothelial cells |
| EMCN | ENSG00000164035 | Endothelial cells |
| ESAM | ENSG00000149564 | Endothelial cells |
| ESM1 | ENSG00000164283 | Endothelial cells |
| HECW2 | ENSG00000138411 | Endothelial cells |
| HHIP | ENSG00000164161 | Endothelial cells |
| KDR | ENSG00000128052 | Endothelial cells |
| MMRN1 | ENSG00000138722 | Endothelial cells |
| MMRN2 | ENSG00000173269 | Endothelial cells |
| MYCT1 | ENSG00000120279 | Endothelial cells |
| PALMD | ENSG00000099260 | Endothelial cells |
| PEAR1 | ENSG00000187800 | Endothelial cells |
| PGF | ENSG00000119630 | Endothelial cells |
| PLXNA2 | ENSG00000076356 | Endothelial cells |
| PTPRB | ENSG00000127329 | Endothelial cells |
| ROBO4 | ENSG00000154133 | Endothelial cells |
| SDPR | ENSG00000168497 | Endothelial cells |
| SHANK3 | ENSG00000251322 | Endothelial cells |
| SHE | ENSG00000169291 | Endothelial cells |
| TEK | ENSG00000120156 | Endothelial cells |
| TIE1 | ENSG00000066056 | Endothelial cells |
| VEPH1 | ENSG00000197415 | Endothelial cells |
| VWF | ENSG00000110799 | Endothelial cells |
| COL1A1 | ENSG00000108821 | Fibroblasts |
| COL3A1 | ENSG00000168542 | Fibroblasts |
| COL6A1 | ENSG00000142156 | Fibroblasts |
| COL6A2 | ENSG00000142173 | Fibroblasts |
| DCN | ENSG00000011465 | Fibroblasts |
| GREM1 | ENSG00000166923 | Fibroblasts |
| PAMR1 | ENSG00000149090 | Fibroblasts |
| TAGLN | ENSG00000149591 | Fibroblasts |
