# Supplementary material for: Identifying and Validating an Acidosis-Related Signature Associated with Prognosis and Tumor Immune Infiltration Characteristics in Pancreatic Carcinoma
Source: J Immunol Res. 2021 Dec 28;2021:3821055. doi: 10.1155/2021/3821055 (PMC8727107; doi:10.1155/2021/3821055)
Supplement: Supplementary Materials — Figure S1: the flow chart of the present study. Figure S2: comparisons of ARI risk groups between different clinical subgroups in TCGA-PAAD. Comparisons of the distribution differences of the acidosis-related index (ARI) risk groups among tumor grade (a), Residual_Tumor (b), Tumor_Status (c), and Progressed (d), respectively. (e) Kaplan–Meier curves and log-rank test of disease-free survival (DFS) outcomes between ARI high-risk and low-risk groups. ARI: acidosis-related index; DFS: disease-free survival. Figure S3: comparisons of the expression levels of the seven key genes in the acidosis-related signature. Figure S4: overall survival analyses of the seven key genes in the acidosis-related signature in TCGA-PAAD. Figure S5: significantly enriched pathways of immunologic signature gene sets in the acidosis-related high-risk group in TCGA-PAAD. Figure S6: correlation analyses between the ARI risk scores and TIDE scores. Pearson correlation analyses between the ARI risk scores and TIDE scores in TCGA-PAAD (a) and GSE62452 (b). ARI: acidosis-related index; TIDE: tumor immune dysfunction and exclusion. Table S1: clinicopathological characteristics of patients enrolled in the present study. Table S2: the specific gene signatures of 24 immune cells. Table S3: the results of the LASSO Cox regression. Table S4: acidosis-related risk scores of patients in TCGA-PAAD. Table S5: acidosis-related risk scores of patients in GSE62452. Table S6: TIDE scores of patients in TCGA-PAAD. Table S7: TIDE scores of patients in GSE62452. [file 3821055.f1.zip › Supplemental Table S3.docx]

| **Table S3 The results of LASSO Cox regression** | |
| --- | --- |
| Gene | Coefficent |
| ARNTL2 | 0.108960189 |
| DKK1 | 0.005561532 |
| CEP55 | 0.120511277 |
| CTSV | 0.096471026 |
| MYEOV | 0.082217227 |
| DSG2 | 0.045503353 |
| GBP2 | 0.05175078 |
