# Supplementary material for: Identifying and Validating an Acidosis-Related Signature Associated with Prognosis and Tumor Immune Infiltration Characteristics in Pancreatic Carcinoma
Source: J Immunol Res. 2021 Dec 28;2021:3821055. doi: 10.1155/2021/3821055 (PMC8727107; doi:10.1155/2021/3821055)
Supplement: Supplementary Materials — Figure S1: the flow chart of the present study. Figure S2: comparisons of ARI risk groups between different clinical subgroups in TCGA-PAAD. Comparisons of the distribution differences of the acidosis-related index (ARI) risk groups among tumor grade (a), Residual_Tumor (b), Tumor_Status (c), and Progressed (d), respectively. (e) Kaplan–Meier curves and log-rank test of disease-free survival (DFS) outcomes between ARI high-risk and low-risk groups. ARI: acidosis-related index; DFS: disease-free survival. Figure S3: comparisons of the expression levels of the seven key genes in the acidosis-related signature. Figure S4: overall survival analyses of the seven key genes in the acidosis-related signature in TCGA-PAAD. Figure S5: significantly enriched pathways of immunologic signature gene sets in the acidosis-related high-risk group in TCGA-PAAD. Figure S6: correlation analyses between the ARI risk scores and TIDE scores. Pearson correlation analyses between the ARI risk scores and TIDE scores in TCGA-PAAD (a) and GSE62452 (b). ARI: acidosis-related index; TIDE: tumor immune dysfunction and exclusion. Table S1: clinicopathological characteristics of patients enrolled in the present study. Table S2: the specific gene signatures of 24 immune cells. Table S3: the results of the LASSO Cox regression. Table S4: acidosis-related risk scores of patients in TCGA-PAAD. Table S5: acidosis-related risk scores of patients in GSE62452. Table S6: TIDE scores of patients in TCGA-PAAD. Table S7: TIDE scores of patients in GSE62452. [file 3821055.f1.zip › Supplemental Table S4.docx]

| **Table S4 Acidosis-related risk scores of 168 patients in TCGA-PAAD** | | | | | | | | | | | |
| --- | --- | --- | --- | --- | --- | --- | --- | --- | --- | --- | --- |
| id | OS time(year) | OS status | ARNTL2 | DKK1 | CEP55 | CTSV | MYEOV | DSG2 | GBP2 | risk score | risk |
| TCGA-IB-7652 | 3.055 | 0 | 3.918881 | 3.402365 | 3.163764 | 2.913916 | 4.858372 | 7.352651 | 5.333779 | 2.118342 | low |
| TCGA-IB-AAUV | 1.105833333 | 0 | 3.290482 | 2.101376 | 2.087725 | 1.878119 | 0.900447 | 5.101834 | 5.497279 | 1.393668 | low |
| TCGA-FB-AAQ3 | 0.085 | 1 | 3.558567 | 3.330652 | 3.82078 | 2.005064 | 5.59373 | 5.763023 | 5.882805 | 2.086721 | low |
| TCGA-IB-7890 | 1.6375 | 1 | 4.449637 | 6.359153 | 4.351266 | 4.015435 | 6.057099 | 8.48184 | 5.080107 | 2.578799 | high |
| TCGA-XD-AAUI | 1.001666667 | 1 | 3.842571 | 5.158838 | 4.006988 | 3.072035 | 5.508217 | 6.743952 | 5.426561 | 2.267199 | high |
| TCGA-2J-AABK | 1.325 | 0 | 2.735012 | 2.272135 | 3.887101 | 1.724505 | 5.797126 | 5.742372 | 4.852707 | 1.9345 | low |
| TCGA-IB-A5SS | 1.259166667 | 1 | 5.165477 | 7.724414 | 5.39316 | 4.820719 | 5.356879 | 7.764501 | 5.782 | 2.813749 | high |
| TCGA-FB-AAPZ | 1.96 | 0 | 4.519159 | 7.775747 | 3.714018 | 3.270384 | 5.414652 | 7.444265 | 6.652283 | 2.42691 | high |
| TCGA-2J-AABI | 2.6525 | 0 | 6.724489 | 4.597104 | 4.64298 | 3.202859 | 3.965737 | 6.237522 | 4.679575 | 2.478835 | high |
| TCGA-HV-A5A6 | 5.574166667 | 1 | 4.741635 | 5.840082 | 4.195407 | 3.583759 | 2.153296 | 5.623269 | 6.127369 | 2.150464 | high |
| TCGA-HZ-8001 | 1.9325 | 0 | 4.086959 | 6.188231 | 3.077372 | 3.537754 | 5.652398 | 5.175026 | 5.956417 | 2.200335 | high |
| TCGA-3E-AAAZ | 5.973333333 | 1 | 3.556073 | 3.466157 | 4.059609 | 1.611857 | 7.412748 | 6.899384 | 5.605856 | 2.264982 | high |
| TCGA-IB-7885 | 3.440833333 | 0 | 4.82506 | 2.923513 | 3.946024 | 3.456904 | 4.142705 | 7.555717 | 6.099456 | 2.351094 | high |
| TCGA-IB-7651 | 1.650833333 | 1 | 4.050892 | 6.692686 | 4.446399 | 2.402724 | 6.448078 | 7.435414 | 6.941915 | 2.473971 | high |
| TCGA-US-A779 | 1.399166667 | 1 | 3.723295 | 5.914524 | 3.245183 | 1.620964 | 6.7127 | 6.810643 | 5.174452 | 2.115631 | low |
| TCGA-HZ-7925 | 1.680833333 | 1 | 4.383408 | 2.39773 | 4.522028 | 2.348368 | 5.671559 | 6.936637 | 5.840955 | 2.346671 | high |
| TCGA-IB-7886 | 0.336666667 | 1 | 5.852072 | 7.358652 | 4.277064 | 2.841482 | 6.543428 | 7.123461 | 7.567413 | 2.721867 | high |
| TCGA-IB-8127 | 1.429166667 | 0 | 5.042054 | 6.676527 | 4.104323 | 2.655707 | 7.65654 | 6.680093 | 5.948126 | 2.578617 | high |
| TCGA-HZ-8637 | 1.415 | 1 | 4.380017 | 2.234427 | 3.984093 | 1.51078 | 2.855227 | 5.55689 | 6.536977 | 1.941448 | low |
| TCGA-HZ-A77P | 0.903333333 | 0 | 3.220045 | 2.417046 | 3.214707 | 1.744519 | 2.542902 | 5.651236 | 6.096145 | 1.701704 | low |
| TCGA-IB-7646 | 0.396666667 | 1 | 5.082525 | 6.928443 | 5.221711 | 3.150078 | 6.668148 | 8.644206 | 6.38227 | 2.797356 | high |
| TCGA-HV-A5A3 | 0.35 | 1 | 5.157839 | 5.977467 | 3.29288 | 2.677584 | 5.286612 | 7.250873 | 6.552165 | 2.354051 | high |
| TCGA-IB-A6UG | 0.1125 | 1 | 4.427652 | 3.617808 | 3.490703 | 2.296873 | 5.228756 | 6.110747 | 5.255675 | 2.124748 | low |
| TCGA-3A-A9J0 | 2.034166667 | 0 | 3.882333 | 4.081524 | 4.228756 | 2.956707 | 3.853264 | 5.955879 | 5.526218 | 2.114372 | low |
| TCGA-IB-AAUQ | 0.500833333 | 1 | 4.30175 | 6.867628 | 3.727142 | 3.139922 | 5.848779 | 8.179966 | 5.4044 | 2.391756 | high |
| TCGA-Q3-AA2A | 0.26 | 0 | 4.353079 | 4.56704 | 3.551276 | 1.525319 | 5.93137 | 5.215326 | 6.162816 | 2.118736 | low |
| TCGA-FB-AAQ2 | 0.419166667 | 1 | 5.238698 | 4.238462 | 5.019521 | 4.836592 | 5.870949 | 7.235711 | 4.765503 | 2.724443 | high |
| TCGA-2L-AAQM | 3.785833333 | 0 | 2.454246 | 0.279714 | 0.411223 | 1.382251 | 0.025642 | 6.932756 | 3.627123 | 0.957153 | low |
| TCGA-IB-7649 | 1.278333333 | 1 | 3.45293 | 4.24628 | 2.754277 | 1.660022 | 3.971102 | 6.89676 | 6.145231 | 1.850252 | low |
| TCGA-HZ-8317 | 1.035 | 1 | 3.595507 | 4.425418 | 3.767663 | 0.938285 | 4.677154 | 7.169267 | 4.670517 | 1.913414 | low |
| TCGA-3A-A9I9 | 1.735833333 | 1 | 2.367541 | 2.970171 | 3.317671 | 1.689101 | 4.909291 | 5.776964 | 4.974521 | 1.761187 | low |
| TCGA-FB-AAPY | 2.899166667 | 1 | 3.621387 | 5.328663 | 2.264857 | 1.871973 | 6.014388 | 5.43068 | 5.827626 | 1.920939 | low |
| TCGA-H6-8124 | 1.073333333 | 0 | 5.182168 | 7.262851 | 3.441459 | 2.788175 | 4.7703 | 7.708336 | 5.84032 | 2.333952 | high |
| TCGA-3E-AAAY | 6.255833333 | 0 | 3.730629 | 4.573517 | 3.236178 | 1.133638 | 4.650749 | 6.138811 | 5.786168 | 1.892432 | low |
| TCGA-HZ-A9TJ | 1.650833333 | 0 | 4.171019 | 4.898337 | 2.606677 | 1.733238 | 1.441239 | 6.791266 | 6.096016 | 1.706052 | low |
| TCGA-LB-A8F3 | 1.0375 | 0 | 2.470227 | 1.959333 | 2.388652 | 1.483936 | 2.061615 | 6.078433 | 4.997561 | 1.415787 | low |
| TCGA-US-A77E | 1.1775 | 1 | 3.87709 | 4.507215 | 3.555027 | 3.062867 | 5.448649 | 7.462092 | 5.902796 | 2.264411 | high |
| TCGA-YY-A8LH | 5.519166667 | 0 | 4.424716 | 5.802155 | 3.840326 | 2.69557 | 6.676823 | 7.454113 | 5.188294 | 2.393869 | high |
| TCGA-IB-7654 | 1.303333333 | 1 | 3.546301 | 0.738563 | 2.628729 | 1.270221 | 4.905955 | 6.844749 | 5.492068 | 1.828876 | low |
| TCGA-FB-A78T | 1.026666667 | 1 | 3.48477 | 1.070869 | 3.375971 | 1.616594 | 7.445796 | 6.152438 | 5.866067 | 2.144157 | high |
| TCGA-2J-AAB8 | 0.219166667 | 0 | 4.009367 | 3.790473 | 4.452389 | 1.476633 | 5.944821 | 5.804147 | 5.44287 | 2.171505 | high |
| TCGA-FB-A4P6 | 2.1 | 0 | 3.601184 | 4.725478 | 2.790224 | 1.51983 | 4.093552 | 7.481167 | 5.894576 | 1.883567 | low |
| TCGA-FB-A7DR | 0.966666667 | 1 | 4.176691 | 7.989896 | 4.636422 | 1.679414 | 6.335126 | 6.317701 | 6.283913 | 2.353815 | high |
| TCGA-2L-AAQL | 0.799166667 | 1 | 3.166596 | 2.359838 | 3.178328 | 1.699488 | 4.908872 | 5.548869 | 5.354628 | 1.838325 | low |
| TCGA-HZ-7920 | 0.645833333 | 1 | 3.804219 | 2.970592 | 2.41828 | 1.704844 | 1.587895 | 7.154097 | 5.586306 | 1.632111 | low |
| TCGA-FB-AAPU | 1.043333333 | 1 | 3.916817 | 4.557046 | 4.27327 | 2.034866 | 7.412225 | 6.67966 | 6.344928 | 2.405119 | high |
| TCGA-IB-A6UF | 1.823333333 | 0 | 3.662509 | 7.175484 | 4.017457 | 2.289724 | 5.796924 | 7.216244 | 6.703141 | 2.295878 | high |
| TCGA-OE-A75W | 0.730833333 | 1 | 3.324534 | 4.422143 | 3.754952 | 2.726043 | 7.38131 | 5.260412 | 5.954435 | 2.256718 | high |
| TCGA-3A-A9IZ | 0.843333333 | 1 | 5.33893 | 7.21338 | 5.069686 | 3.256534 | 6.485482 | 7.619204 | 6.699544 | 2.773588 | high |
| TCGA-2J-AABU | 0.758333333 | 1 | 5.611506 | 7.471219 | 3.921765 | 4.307289 | 6.246622 | 7.106549 | 6.543596 | 2.716716 | high |
| TCGA-HZ-7924 | 2.3 | 0 | 4.065883 | 5.161771 | 4.529194 | 1.757049 | 3.362141 | 8.128284 | 5.87198 | 2.13722 | high |
| TCGA-HZ-7289 | 1.809166667 | 1 | 5.323188 | 1.136158 | 4.664207 | 3.369613 | 2.820075 | 6.906747 | 3.910338 | 2.221996 | high |
| TCGA-HZ-7926 | 1.418333333 | 1 | 4.751342 | 7.207184 | 3.99803 | 3.094161 | 4.698606 | 8.136234 | 5.815677 | 2.395593 | high |
| TCGA-RB-AA9M | 0.783333333 | 0 | 3.945668 | 4.127554 | 4.334634 | 2.708731 | 4.556332 | 7.333621 | 5.45187 | 2.227014 | high |
| TCGA-2J-AABH | 3.523333333 | 0 | 3.894339 | 4.149245 | 3.252956 | 2.076151 | 1.966246 | 7.029865 | 5.083992 | 1.784353 | low |
| TCGA-IB-7645 | 4.111666667 | 1 | 4.19915 | 4.028742 | 3.173688 | 1.398198 | 5.097747 | 7.354912 | 6.016597 | 2.062456 | low |
| TCGA-2J-AABA | 1.661666667 | 1 | 4.150486 | 3.473112 | 2.727312 | 4.054131 | 6.284881 | 6.647776 | 6.088636 | 2.325645 | high |
| TCGA-HZ-A4BH | 0.530833333 | 0 | 3.368762 | 3.354009 | 4.228164 | 2.364209 | 7.224769 | 7.06275 | 6.084751 | 2.353603 | high |
| TCGA-FB-AAPQ | 3.093333333 | 1 | 3.293008 | 1.377266 | 3.983336 | 2.041551 | 5.288439 | 7.925971 | 5.248439 | 2.110524 | low |
| TCGA-FB-AAQ0 | 1.295 | 1 | 4.140094 | 6.782443 | 4.141158 | 0.718353 | 6.069598 | 6.85396 | 6.663908 | 2.212949 | high |
| TCGA-2J-AAB1 | 0.180833333 | 1 | 3.711615 | 6.813349 | 3.643266 | 3.55171 | 8.164307 | 6.935972 | 6.312417 | 2.537532 | high |
| TCGA-H6-A45N | 1.1525 | 1 | 3.318141 | 3.853004 | 3.524416 | 2.084671 | 5.049333 | 6.578509 | 5.747287 | 2.020729 | low |
| TCGA-F2-A44H | 1.604166667 | 0 | 3.074993 | 0.575098 | 2.826958 | 0.738769 | 0.702174 | 5.63468 | 4.529732 | 1.298745 | low |
| TCGA-IB-7644 | 1.078333333 | 1 | 4.519583 | 3.251764 | 4.721371 | 2.630986 | 6.024286 | 7.92354 | 6.715388 | 2.536706 | high |
| TCGA-3A-A9IB | 0.613333333 | 1 | 5.514455 | 4.890219 | 4.673012 | 1.499609 | 5.489741 | 7.706488 | 6.670241 | 2.483085 | high |
| TCGA-IB-AAUW | 0.63 | 1 | 3.654706 | 4.27721 | 2.16786 | 1.528227 | 4.463475 | 7.291957 | 5.945209 | 1.837139 | low |
| TCGA-HZ-7919 | 1.623333333 | 1 | 4.818665 | 7.765614 | 4.928594 | 1.793347 | 6.378204 | 7.932643 | 6.647017 | 2.564537 | high |
| TCGA-FB-AAPS | 0.624166667 | 0 | 3.716386 | 2.866048 | 3.14673 | 0.934549 | 3.305257 | 5.528437 | 5.789816 | 1.71319 | low |
| TCGA-LB-A7SX | 1.075833333 | 1 | 3.521064 | 6.245709 | 4.882333 | 2.439661 | 4.434438 | 5.67045 | 4.663695 | 2.106086 | low |
| TCGA-IB-AAUS | 0.615833333 | 0 | 3.939472 | 4.300486 | 3.636588 | 1.444952 | 3.562909 | 5.596965 | 5.597139 | 1.868078 | low |
| TCGA-HV-A7OP | 2.6775 | 0 | 1.176352 | 0 | 3.11454 | 0.756114 | 0.57216 | 4.856713 | 4.914096 | 1.098802 | low |
| TCGA-FB-AAQ1 | 0.336666667 | 1 | 4.54554 | 3.119168 | 4.727197 | 1.777996 | 6.182528 | 7.48507 | 4.95514 | 2.359174 | high |
| TCGA-IB-7888 | 3.646666667 | 1 | 3.420627 | 4.084108 | 3.229639 | 0.958253 | 5.130492 | 6.855922 | 5.812125 | 1.911642 | low |
| TCGA-F2-7273 | 1.620833333 | 1 | 3.82209 | 4.409588 | 2.678133 | 2.273379 | 2.626227 | 7.181395 | 5.92608 | 1.832418 | low |
| TCGA-2L-AAQI | 0.281666667 | 1 | 4.466125 | 3.97037 | 3.919309 | 5.266145 | 6.246545 | 7.564811 | 5.343595 | 2.623396 | high |
| TCGA-XD-AAUG | 1.15 | 0 | 2.900443 | 2.603232 | 1.510892 | 1.080308 | 3.549981 | 5.170564 | 5.289276 | 1.417681 | low |
| TCGA-3A-A9IS | 2.7325 | 0 | 3.969693 | 0 | 1.295401 | 0.60462 | 0 | 5.945047 | 1.24812 | 0.982088 | low |
| TCGA-IB-AAUT | 0.785833333 | 0 | 3.467378 | 0.672323 | 2.134631 | 2.218774 | 1.246509 | 6.665924 | 6.247845 | 1.581977 | low |
| TCGA-Z5-AAPL | 1.278333333 | 0 | 3.688238 | 3.065944 | 4.345042 | 1.142607 | 3.512287 | 5.157216 | 5.90677 | 1.881899 | low |
| TCGA-HZ-A4BK | 1.798333333 | 0 | 3.784137 | 6.579443 | 3.843886 | 2.365384 | 5.320497 | 6.218242 | 6.552935 | 2.199842 | high |
| TCGA-US-A776 | 3.329166667 | 0 | 4.603539 | 0.267559 | 4.605553 | 1.086641 | 4.994322 | 5.271503 | 5.513358 | 2.098752 | low |
| TCGA-IB-7889 | 1.316666667 | 1 | 3.784834 | 2.812247 | 3.12637 | 3.194743 | 3.806698 | 6.327372 | 6.15179 | 2.032252 | low |
| TCGA-IB-AAUR | 0.925 | 0 | 3.596236 | 4.056353 | 2.572048 | 1.265779 | 5.104764 | 4.652632 | 5.738412 | 1.774855 | low |
| TCGA-IB-A7M4 | 1.3225 | 0 | 4.05699 | 8.200812 | 4.567088 | 3.826 | 6.622141 | 8.849718 | 6.152212 | 2.672671 | high |
| TCGA-IB-A5ST | 1.738333333 | 0 | 3.371521 | 2.51792 | 3.625076 | 0.693409 | 3.991164 | 5.147467 | 5.492525 | 1.731734 | low |
| TCGA-3A-A9IU | 1.254166667 | 1 | 5.215576 | 4.261003 | 3.018853 | 5.122111 | 5.867364 | 7.416966 | 6.303697 | 2.596045 | high |
| TCGA-3A-A9IJ | 5.075833333 | 0 | 1.74481 | 0.126462 | 0.226397 | 1.414809 | 0.019257 | 2.709781 | 1.992289 | 0.582579 | low |
| TCGA-3A-A9IH | 2.795 | 0 | 4.525364 | 4.102521 | 4.108994 | 2.406805 | 6.615056 | 6.873228 | 5.93362 | 2.406964 | high |
| TCGA-F2-A8YN | 1.415 | 0 | 3.522099 | 4.350195 | 3.775258 | 2.307387 | 5.421754 | 6.772499 | 6.549048 | 2.178371 | high |
| TCGA-HZ-A49I | 0.843333333 | 1 | 3.932897 | 2.674792 | 2.691583 | 3.380643 | 6.574758 | 6.824132 | 5.768491 | 2.243508 | high |
| TCGA-HV-A5A4 | 0.635 | 0 | 3.997381 | 1.466247 | 3.377556 | 1.601999 | 6.319032 | 6.860859 | 6.345412 | 2.165396 | high |
| TCGA-HZ-8636 | 1.491666667 | 1 | 5.315821 | 4.934403 | 4.226934 | 2.221102 | 4.031006 | 7.583227 | 5.920237 | 2.313178 | high |
| TCGA-2J-AABO | 1.204166667 | 0 | 4.355006 | 6.892068 | 3.376695 | 2.716665 | 6.766498 | 5.928919 | 5.797093 | 2.307975 | high |
| TCGA-FB-A5VM | 1.363333333 | 1 | 4.92584 | 4.243089 | 5.414144 | 5.017346 | 6.112223 | 4.770075 | 5.666738 | 2.709655 | high |
| TCGA-HZ-8315 | 0.818333333 | 1 | 4.417515 | 5.787054 | 3.356663 | 2.420129 | 5.963491 | 7.068515 | 6.516582 | 2.300687 | high |
| TCGA-F2-6880 | 0.8075 | 0 | 0.412273 | 0 | 0.145252 | 0.167183 | 2.183511 | 4.258644 | 1.751784 | 0.542515 | low |
| TCGA-2L-AAQJ | 1.078333333 | 1 | 3.437022 | 5.274667 | 3.620308 | 2.716971 | 6.070272 | 6.602978 | 5.641237 | 2.193708 | high |
| TCGA-HZ-8005 | 0.328333333 | 1 | 5.690779 | 3.255521 | 4.438493 | 2.150496 | 5.858483 | 8.235671 | 7.249126 | 2.61209 | high |
| TCGA-2J-AAB9 | 1.716666667 | 1 | 4.465904 | 4.548355 | 3.042339 | 2.55416 | 4.956389 | 7.35404 | 5.269844 | 2.139793 | high |
| TCGA-XN-A8T5 | 1.970833333 | 0 | 4.2149 | 5.432209 | 2.851326 | 0.726335 | 3.592904 | 4.684867 | 5.719117 | 1.707699 | low |
| TCGA-HZ-8638 | 0.413333333 | 1 | 4.451098 | 4.46323 | 4.556111 | 1.334883 | 3.602015 | 7.713229 | 6.675414 | 2.180239 | high |
| TCGA-L1-A7W4 | 0.760833333 | 1 | 5.186871 | 5.317977 | 5.692425 | 6.804664 | 5.852252 | 7.363043 | 5.34377 | 3.029936 | high |
| TCGA-3A-A9IX | 2.839166667 | 0 | 3.332362 | 3.562837 | 2.499856 | 1.709053 | 4.138481 | 6.120319 | 5.479347 | 1.751354 | low |
| TCGA-XD-AAUH | 1.081666667 | 0 | 3.240251 | 3.222368 | 2.32511 | 1.265222 | 4.516181 | 5.602897 | 5.032796 | 1.659949 | low |
| TCGA-HZ-A77O | 0.438333333 | 1 | 4.40535 | 4.233774 | 3.856019 | 2.058776 | 5.523193 | 7.436741 | 4.722375 | 2.203745 | high |
| TCGA-IB-A5SO | 0.999166667 | 1 | 3.618873 | 3.979934 | 3.430778 | 2.297247 | 5.325484 | 6.389593 | 5.962674 | 2.08868 | low |
| TCGA-F2-6879 | 0.914166667 | 1 | 4.452892 | 6.710285 | 5.299183 | 2.672913 | 6.04507 | 6.900354 | 6.527796 | 2.567794 | high |
| TCGA-2J-AAB4 | 1.995833333 | 0 | 4.031645 | 2.906311 | 4.516638 | 2.075637 | 6.591775 | 6.883716 | 5.985427 | 2.364937 | high |
| TCGA-US-A774 | 1.9025 | 1 | 3.867554 | 4.097632 | 3.265034 | 2.1728 | 6.035072 | 6.517219 | 5.685828 | 2.134272 | low |
| TCGA-IB-8126 | 1.265 | 0 | 1.96675 | 1.554203 | 1.454978 | 0.475831 | 2.632953 | 5.393178 | 4.378201 | 1.132643 | low |
| TCGA-F2-A44G | 0.6375 | 1 | 5.155756 | 6.172051 | 4.70698 | 1.737674 | 5.391587 | 7.986442 | 6.013275 | 2.44886 | high |
| TCGA-2J-AABR | 1.199166667 | 0 | 4.27621 | 3.177887 | 3.168847 | 2.47423 | 4.644874 | 6.987334 | 6.138391 | 2.121686 | low |
| TCGA-IB-A5SP | 1.319166667 | 0 | 2.790367 | 2.290306 | 4.131388 | 1.645654 | 5.938379 | 6.706441 | 5.452263 | 2.048975 | low |
| TCGA-H8-A6C1 | 1.836666667 | 0 | 4.088919 | 4.817617 | 3.936749 | 1.245491 | 5.86251 | 5.826906 | 4.591309 | 2.051646 | low |
| TCGA-2J-AABE | 1.850833333 | 0 | 3.606725 | 5.118348 | 2.934649 | 1.376358 | 3.534548 | 6.283073 | 5.919689 | 1.790742 | low |
| TCGA-RB-A7B8 | 1.275833333 | 1 | 3.095424 | 4.037142 | 3.050968 | 1.731541 | 6.276776 | 5.87593 | 5.647703 | 1.970157 | low |
| TCGA-IB-AAUO | 0.654166667 | 1 | 4.75595 | 5.457857 | 4.438677 | 3.144048 | 7.407981 | 6.980449 | 6.610587 | 2.655584 | high |
| TCGA-2J-AAB6 | 0.8025 | 1 | 5.950426 | 7.00602 | 4.748387 | 3.1301 | 7.285342 | 6.712837 | 5.487574 | 2.749945 | high |
| TCGA-2J-AABF | 1.891666667 | 1 | 5.298979 | 3.837737 | 4.436383 | 1.644088 | 6.132641 | 6.913439 | 6.032954 | 2.422966 | high |
| TCGA-HZ-8002 | 1.001666667 | 1 | 4.461514 | 5.0277 | 3.874548 | 1.907814 | 3.113834 | 7.742176 | 5.884554 | 2.077901 | low |
| TCGA-FB-AAQ6 | 0.668333333 | 1 | 3.760132 | 2.499347 | 3.405887 | 0.898137 | 5.822679 | 6.643877 | 5.834905 | 2.003701 | low |
| TCGA-IB-7891 | 2.499166667 | 1 | 4.248418 | 5.571964 | 3.245291 | 1.383515 | 3.832218 | 6.783163 | 5.953703 | 1.9503 | low |
| TCGA-YB-A89D | 0.958333333 | 0 | 3.981113 | 4.987476 | 3.149156 | 1.622213 | 6.012624 | 7.178975 | 6.028223 | 2.1305 | low |
| TCGA-IB-A5SQ | 0.599166667 | 1 | 4.448168 | 5.527847 | 4.365264 | 4.213275 | 6.1367 | 6.449728 | 5.721484 | 2.542057 | high |
| TCGA-2J-AABP | 1.2675 | 0 | 3.235108 | 2.626497 | 4.806582 | 2.227849 | 2.705414 | 3.798105 | 5.964822 | 1.865218 | low |
| TCGA-IB-A7LX | 0.684166667 | 1 | 6.326976 | 3.425183 | 4.859231 | 4.165165 | 4.541114 | 8.241354 | 5.650503 | 2.736633 | high |
| TCGA-US-A77J | 1.555 | 1 | 3.160915 | 2.858386 | 2.750743 | 2.448489 | 1.753211 | 5.37375 | 5.675111 | 1.610374 | low |
| TCGA-HV-AA8X | 1.456666667 | 1 | 3.708524 | 1.033794 | 4.552085 | 2.495384 | 1.382494 | 6.582273 | 6.051268 | 1.925479 | low |
| TCGA-HZ-A77Q | 0.09 | 0 | 4.111595 | 4.790405 | 2.642096 | 1.38384 | 4.779748 | 5.755285 | 6.114931 | 1.89786 | low |
| TCGA-2J-AABV | 1.785 | 1 | 1.58139 | 1.899661 | 1.209092 | 0.44327 | 2.34184 | 2.969856 | 2.737771 | 0.840705 | low |
| TCGA-LB-A9Q5 | 0.856666667 | 1 | 3.634756 | 4.536204 | 2.128434 | 0.644591 | 4.076562 | 5.958782 | 4.123131 | 1.55964 | low |
| TCGA-S4-A8RO | 1.4375 | 0 | 4.27777 | 2.562217 | 4.443546 | 2.494943 | 7.721425 | 6.505478 | 4.506743 | 2.420626 | high |
| TCGA-3A-A9IC | 2.02 | 1 | 3.596121 | 4.068314 | 4.731811 | 1.882595 | 4.849037 | 6.422723 | 5.681564 | 2.151268 | high |
| TCGA-HZ-8519 | 1.2425 | 0 | 2.359139 | 0.661575 | 3.318037 | 0.601613 | 4.115267 | 6.302088 | 5.109242 | 1.60815 | low |
| TCGA-3A-A9IR | 4.221666667 | 0 | 1.058512 | 0.074697 | 0.363679 | 1.312682 | 0.044292 | 6.784921 | 2.454919 | 0.725636 | low |
| TCGA-HV-AA8V | 2.518333333 | 0 | 3.484945 | 4.802961 | 3.104644 | 3.198651 | 4.631765 | 6.34104 | 5.468352 | 2.041495 | low |
| TCGA-Q3-A5QY | 1.139166667 | 0 | 2.70904 | 3.394163 | 2.723107 | 1.293672 | 4.010314 | 4.107874 | 5.355877 | 1.560831 | low |
| TCGA-3A-A9IL | 7.504166667 | 0 | 1.931745 | 0.189738 | 0.596345 | 0.586295 | 0.199671 | 5.214363 | 3.200284 | 0.75927 | low |
| TCGA-HZ-A49G | 1.806666667 | 0 | 3.639373 | 2.878339 | 3.187829 | 1.661979 | 6.021706 | 6.730409 | 5.512191 | 2.043661 | low |
| TCGA-S4-A8RP | 1.921666667 | 1 | 3.848702 | 4.157363 | 3.413565 | 1.825231 | 6.718684 | 6.576139 | 6.56921 | 2.221521 | high |
| TCGA-HZ-A49H | 1.344166667 | 0 | 3.09895 | 2.533864 | 2.777727 | 0.989945 | 3.886011 | 4.940314 | 5.276893 | 1.599384 | low |
| TCGA-S4-A8RM | 2.0175 | 0 | 4.122071 | 1.98489 | 3.015686 | 3.124798 | 7.321355 | 7.206596 | 5.572136 | 2.343286 | high |
| TCGA-IB-AAUP | 1.18 | 0 | 3.664137 | 3.992074 | 3.852836 | 2.858624 | 4.738106 | 5.232023 | 5.782723 | 2.088421 | low |
| TCGA-IB-AAUN | 0.394166667 | 1 | 4.664072 | 6.072101 | 5.134125 | 2.85785 | 5.022146 | 6.693167 | 6.086177 | 2.468821 | high |
| TCGA-HZ-7918 | 2.6525 | 0 | 3.878666 | 2.313793 | 3.44904 | 2.59464 | 2.475068 | 7.072339 | 6.209506 | 1.948099 | low |
| TCGA-IB-7887 | 0.300833333 | 1 | 5.027098 | 3.432524 | 3.1615 | 1.869684 | 6.227732 | 7.678239 | 6.939536 | 2.348749 | high |
| TCGA-3A-A9IN | 5.705 | 0 | 2.224439 | 0.632385 | 0.684468 | 0.578158 | 0.102127 | 4.665229 | 3.542606 | 0.788167 | low |
| TCGA-F2-7276 | 0.591666667 | 1 | 4.405156 | 3.956481 | 2.785864 | 2.548148 | 2.931871 | 7.405747 | 5.827838 | 1.963173 | low |
| TCGA-HV-A5A5 | 0.790833333 | 0 | 3.562759 | 4.117761 | 3.41624 | 0.800524 | 5.713335 | 6.36511 | 5.2258 | 1.929831 | low |
| TCGA-IB-7893 | 0.32 | 1 | 5.095791 | 5.715088 | 4.926432 | 4.432675 | 6.592825 | 7.182573 | 6.006944 | 2.788077 | high |
| TCGA-2L-AAQE | 1.8725 | 1 | 4.609951 | 6.532649 | 4.241122 | 3.064597 | 6.523216 | 6.160013 | 6.656986 | 2.506507 | high |
| TCGA-HZ-8003 | 1.631666667 | 1 | 3.070406 | 4.105562 | 3.273154 | 0.990162 | 2.524021 | 5.925917 | 4.438543 | 1.554224 | low |
| TCGA-M8-A5N4 | 1.599166667 | 0 | 4.451895 | 6.61378 | 4.079867 | 1.514051 | 6.557686 | 7.279723 | 6.431116 | 2.362816 | high |
| TCGA-2J-AABT | 0.873333333 | 0 | 3.743794 | 2.998046 | 2.829691 | 1.119621 | 4.531649 | 8.439659 | 6.580429 | 1.970774 | low |
| TCGA-IB-AAUU | 0.670833333 | 0 | 4.546917 | 5.078742 | 4.493277 | 2.424734 | 6.510041 | 6.397534 | 6.263949 | 2.449597 | high |
| TCGA-FB-A545 | 2.004166667 | 1 | 5.742164 | 6.815254 | 4.72429 | 3.747628 | 6.750322 | 7.493189 | 5.445852 | 2.772223 | high |
| TCGA-PZ-A5RE | 1.286666667 | 1 | 3.870922 | 6.817724 | 3.697388 | 2.837746 | 6.428509 | 6.201889 | 6.087918 | 2.304826 | high |
| TCGA-HV-A7OL | 0.69 | 0 | 3.460194 | 4.593621 | 3.589282 | 2.888996 | 5.951291 | 5.194306 | 6.068897 | 2.153551 | high |
| TCGA-IB-7897 | 1.330833333 | 1 | 4.271191 | 2.315874 | 3.642989 | 1.499282 | 2.450498 | 6.908053 | 6.238452 | 1.900585 | low |
| TCGA-XN-A8T3 | 2.603333333 | 0 | 4.143935 | 1.392603 | 4.388078 | 2.547931 | 5.255279 | 6.878301 | 5.904444 | 2.284503 | high |
| TCGA-HZ-7923 | 0.86 | 0 | 3.418973 | 1.577747 | 3.021528 | 1.088297 | 2.395543 | 6.076612 | 6.275226 | 1.648633 | low |
| TCGA-FB-A4P5 | 0.49 | 1 | 3.364999 | 6.087431 | 3.941646 | 1.599079 | 5.955247 | 6.100652 | 5.571547 | 2.08534 | low |
| TCGA-2L-AAQA | 0.391666667 | 1 | 4.170193 | 2.991918 | 4.926347 | 2.85355 | 6.915536 | 7.002976 | 5.031106 | 2.487589 | high |
| TCGA-3A-A9IV | 3.02 | 0 | 2.696767 | 0.293282 | 2.441101 | 2.056257 | 0.027004 | 6.623489 | 2.982743 | 1.245991 | low |
| TCGA-3A-A9I7 | 3.621666667 | 0 | 4.074202 | 3.410172 | 3.444407 | 1.822146 | 4.557216 | 6.969447 | 5.640858 | 2.0375 | low |
| TCGA-FB-AAPP | 1.3275 | 1 | 4.443378 | 0.171764 | 5.299065 | 3.165403 | 7.236429 | 7.365269 | 4.805355 | 2.607858 | high |
| TCGA-F2-A7TX | 0.26 | 1 | 5.418469 | 7.158807 | 5.404807 | 3.424702 | 5.110783 | 7.011529 | 6.534744 | 2.689357 | high |
| TCGA-XD-AAUL | 1.363333333 | 0 | 4.929504 | 5.590952 | 3.969302 | 2.234918 | 6.711623 | 7.354207 | 5.862259 | 2.451993 | high |
