# Supplementary material for: Identifying and Validating an Acidosis-Related Signature Associated with Prognosis and Tumor Immune Infiltration Characteristics in Pancreatic Carcinoma
Source: J Immunol Res. 2021 Dec 28;2021:3821055. doi: 10.1155/2021/3821055 (PMC8727107; doi:10.1155/2021/3821055)
Supplement: Supplementary Materials — Figure S1: the flow chart of the present study. Figure S2: comparisons of ARI risk groups between different clinical subgroups in TCGA-PAAD. Comparisons of the distribution differences of the acidosis-related index (ARI) risk groups among tumor grade (a), Residual_Tumor (b), Tumor_Status (c), and Progressed (d), respectively. (e) Kaplan–Meier curves and log-rank test of disease-free survival (DFS) outcomes between ARI high-risk and low-risk groups. ARI: acidosis-related index; DFS: disease-free survival. Figure S3: comparisons of the expression levels of the seven key genes in the acidosis-related signature. Figure S4: overall survival analyses of the seven key genes in the acidosis-related signature in TCGA-PAAD. Figure S5: significantly enriched pathways of immunologic signature gene sets in the acidosis-related high-risk group in TCGA-PAAD. Figure S6: correlation analyses between the ARI risk scores and TIDE scores. Pearson correlation analyses between the ARI risk scores and TIDE scores in TCGA-PAAD (a) and GSE62452 (b). ARI: acidosis-related index; TIDE: tumor immune dysfunction and exclusion. Table S1: clinicopathological characteristics of patients enrolled in the present study. Table S2: the specific gene signatures of 24 immune cells. Table S3: the results of the LASSO Cox regression. Table S4: acidosis-related risk scores of patients in TCGA-PAAD. Table S5: acidosis-related risk scores of patients in GSE62452. Table S6: TIDE scores of patients in TCGA-PAAD. Table S7: TIDE scores of patients in GSE62452. [file 3821055.f1.zip › Supplemental Table S5.docx]

| **Table S5 Acidosis-related risk scores of 63 patients in GSE62452** | | | | | | | | | | | |
| --- | --- | --- | --- | --- | --- | --- | --- | --- | --- | --- | --- |
| id | OS time(year) | OS status | ARNTL2 | DKK1 | CEP55 | CTSV | MYEOV | DSG2 | GBP2 | risk score | risk |
| GSM1527183 | 0.35 | 1 | 5.634595 | 5.139667 | 5.111921 | 4.797983 | 5.759421 | 6.956071 | 5.905933 | 2.817126 | high |
| GSM1527196 | 0.791666667 | 1 | 5.942464 | 4.248779 | 4.006067 | 3.895432 | 6.238349 | 7.020183 | 5.746432 | 2.659418 | high |
| GSM1527207 | 1.183333333 | 1 | 5.848596 | 5.29036 | 4.757208 | 3.654255 | 5.798476 | 7.764599 | 5.795809 | 2.722501 | high |
| GSM1527232 | 4.141666667 | 1 | 4.767239 | 3.366209 | 3.240381 | 3.296616 | 4.356846 | 6.952965 | 5.348783 | 2.198086 | low |
| GSM1527105 | 4.258333333 | 1 | 4.934086 | 3.637446 | 3.48879 | 3.390173 | 5.870202 | 6.602542 | 5.158725 | 2.355378 | low |
| GSM1527109 | 0.225 | 1 | 6.734172 | 4.884285 | 4.709953 | 4.448763 | 5.879772 | 7.351198 | 4.613418 | 2.814371 | high |
| GSM1527137 | 3.333333333 | 0 | 4.012321 | 3.081557 | 2.645951 | 3.15758 | 4.663 | 6.271046 | 4.602496 | 1.984719 | low |
| GSM1527141 | 1.1 | 1 | 5.776952 | 4.072572 | 4.202199 | 3.718028 | 5.043025 | 7.522698 | 5.29444 | 2.548125 | low |
| GSM1527151 | 2.3 | 0 | 4.999368 | 3.702894 | 3.417027 | 4.01394 | 4.918969 | 6.894758 | 5.550711 | 2.369757 | low |
| GSM1527167 | 1.766666667 | 0 | 6.139266 | 4.561217 | 4.343454 | 3.636602 | 5.414616 | 6.957788 | 5.779221 | 2.629421 | high |
| GSM1527209 | 2.666666667 | 1 | 6.317734 | 5.744311 | 4.636186 | 4.208197 | 5.348315 | 7.657134 | 5.757388 | 2.771109 | high |
| GSM1527212 | 3.825 | 1 | 4.983957 | 4.004158 | 3.416099 | 3.402109 | 4.669403 | 6.014666 | 5.668547 | 2.25615 | low |
| GSM1527218 | 1.141666667 | 1 | 7.549247 | 5.290891 | 5.639374 | 4.587136 | 5.645126 | 7.587263 | 5.719782 | 3.079502 | high |
| GSM1527230 | 5.641666667 | 0 | 4.546558 | 3.275974 | 3.736908 | 3.344129 | 4.184377 | 4.552246 | 6.472368 | 2.172685 | low |
| GSM1527139 | 0.1 | 1 | 3.762414 | 2.759209 | 2.424313 | 3.046729 | 4.06743 | 6.574895 | 4.526461 | 1.879217 | low |
| GSM1527145 | 0.9 | 1 | 5.593954 | 3.657199 | 4.548093 | 3.042282 | 4.890824 | 7.058516 | 6.227327 | 2.517012 | low |
| GSM1527147 | 2.416666667 | 1 | 4.860069 | 3.043301 | 3.302174 | 3.11192 | 4.498588 | 6.097614 | 5.478768 | 2.175493 | low |
| GSM1527149 | 2.308333333 | 1 | 5.080137 | 3.961235 | 3.601935 | 3.107703 | 4.582629 | 6.646212 | 5.810345 | 2.289326 | low |
| GSM1527155 | 0.566666667 | 1 | 5.892729 | 5.888171 | 4.460879 | 3.608619 | 5.385299 | 7.694033 | 6.023537 | 2.665125 | high |
| GSM1527157 | 2.35 | 0 | 5.638811 | 4.398779 | 3.988495 | 3.240069 | 5.259662 | 6.563728 | 6.230559 | 2.485644 | low |
| GSM1527159 | 0.816666667 | 1 | 6.022343 | 5.187049 | 5.062723 | 3.163716 | 4.638073 | 6.192326 | 6.718661 | 2.611163 | high |
| GSM1527161 | 1.966666667 | 0 | 5.455241 | 5.218756 | 3.688665 | 4.123734 | 4.249615 | 6.260634 | 5.735484 | 2.396862 | low |
| GSM1527163 | 0.641666667 | 1 | 6.740871 | 6.413256 | 4.947096 | 3.598548 | 5.348979 | 6.930368 | 5.185664 | 2.736986 | high |
| GSM1527165 | 1.816666667 | 0 | 5.414259 | 4.813611 | 4.603917 | 3.77998 | 5.858966 | 6.873743 | 5.480141 | 2.61428 | high |
| GSM1527169 | 1.758333333 | 0 | 6.156797 | 4.439694 | 4.610358 | 4.470927 | 5.492784 | 6.644146 | 6.133042 | 2.753774 | high |
| GSM1527171 | 0.741666667 | 1 | 5.471448 | 5.809701 | 4.159712 | 3.286129 | 5.263898 | 8.293335 | 7.004958 | 2.619459 | high |
| GSM1527175 | 0.533333333 | 1 | 6.426689 | 4.602362 | 4.557827 | 3.634093 | 5.669273 | 6.213066 | 5.767072 | 2.672981 | high |
| GSM1527177 | 1.366666667 | 0 | 6.047798 | 4.177465 | 3.928597 | 4.673957 | 4.346162 | 7.588617 | 5.233471 | 2.580017 | high |
| GSM1527179 | 0.383333333 | 1 | 5.541679 | 4.258583 | 3.872777 | 3.946483 | 4.786413 | 7.059516 | 5.33573 | 2.465827 | low |
| GSM1527181 | 0.883333333 | 0 | 5.563469 | 4.404889 | 3.949711 | 4.661909 | 5.804923 | 7.85314 | 6.029964 | 2.703083 | high |
| GSM1527185 | 0.858333333 | 1 | 3.738065 | 2.936218 | 2.841739 | 3.229939 | 4.79141 | 5.99662 | 4.312754 | 1.967678 | low |
| GSM1527189 | 1.241666667 | 1 | 5.47759 | 3.807324 | 3.911109 | 4.819142 | 5.273754 | 6.73835 | 5.839528 | 2.596665 | high |
| GSM1527191 | 0.375 | 1 | 4.538936 | 4.041907 | 3.593352 | 3.554585 | 4.584848 | 4.179425 | 5.518008 | 2.145689 | low |
| GSM1527193 | 1.075 | 1 | 5.169388 | 3.478894 | 3.589676 | 3.844877 | 4.963704 | 6.731616 | 5.339802 | 2.376873 | low |
| GSM1527198 | 0.525 | 1 | 6.27162 | 4.405878 | 4.272727 | 4.696073 | 5.461332 | 7.935745 | 5.609769 | 2.776236 | high |
| GSM1527200 | 0.491666667 | 1 | 4.553677 | 2.935319 | 3.596687 | 3.171875 | 3.825274 | 3.636265 | 6.344565 | 2.060232 | low |
| GSM1527202 | 0.816666667 | 1 | 5.367107 | 3.731708 | 4.308344 | 4.004881 | 5.615517 | 7.131971 | 6.018584 | 2.608801 | high |
| GSM1527204 | 0.441666667 | 1 | 6.538125 | 6.409534 | 5.10441 | 3.544097 | 5.506655 | 7.312111 | 6.362207 | 2.819801 | high |
| GSM1527205 | 1.791666667 | 1 | 4.839418 | 3.264585 | 3.399349 | 3.256265 | 4.591053 | 7.259672 | 5.450756 | 2.259139 | low |
| GSM1527213 | 1.825 | 1 | 6.081235 | 2.994111 | 4.680351 | 3.517045 | 5.394796 | 7.230879 | 5.805339 | 2.655598 | high |
| GSM1527215 | 3.5 | 0 | 4.755192 | 3.370722 | 3.353554 | 3.67747 | 4.183408 | 5.788488 | 5.62972 | 2.19447 | low |
| GSM1527216 | 3.191666667 | 0 | 4.562613 | 3.573358 | 3.545381 | 3.683453 | 4.737451 | 6.363947 | 5.275259 | 2.251701 | low |
| GSM1527219 | 0.908333333 | 1 | 6.056386 | 5.288615 | 3.886588 | 3.502563 | 5.120173 | 6.020917 | 6.624044 | 2.533329 | low |
| GSM1527220 | 1.775 | 1 | 6.57237 | 6.061812 | 4.890408 | 3.617537 | 4.371983 | 9.698141 | 5.947869 | 2.796734 | high |
| GSM1527223 | 0.775 | 1 | 5.128225 | 4.039051 | 3.321136 | 3.210342 | 4.859497 | 6.681722 | 5.12666 | 2.260059 | low |
| GSM1527225 | 1.658333333 | 1 | 5.62558 | 4.657376 | 3.587483 | 3.714368 | 5.601771 | 7.112068 | 4.851856 | 2.4648 | low |
| GSM1527227 | 5.9 | 0 | 4.064386 | 3.590348 | 3.29215 | 3.489603 | 5.815574 | 6.102423 | 3.572009 | 2.136886 | low |
| GSM1527107 | 0.575 | 1 | 6.924944 | 3.694406 | 4.918659 | 3.716137 | 6.145961 | 9.46682 | 6.599315 | 3.003939 | high |
| GSM1527111 | 3.466666667 | 1 | 4.449221 | 2.93382 | 2.882781 | 2.846077 | 4.626732 | 4.405181 | 6.513622 | 2.041009 | low |
| GSM1527115 | 2.991666667 | 1 | 5.750496 | 3.709848 | 4.98065 | 3.197749 | 5.080967 | 8.236503 | 5.625381 | 2.639572 | high |
| GSM1527117 | 0.2 | 1 | 5.501984 | 4.585072 | 3.999061 | 4.239109 | 5.099161 | 8.219852 | 5.371167 | 2.587112 | high |
| GSM1527125 | 1.05 | 1 | 6.524538 | 5.424491 | 3.801488 | 5.645962 | 5.307952 | 6.406931 | 6.120014 | 2.788535 | high |
| GSM1527129 | 3.408333333 | 1 | 4.982452 | 3.996006 | 4.040116 | 3.957738 | 6.095989 | 6.82262 | 5.927322 | 2.55219 | low |
| GSM1527133 | 0.966666667 | 1 | 6.410988 | 6.705921 | 5.682817 | 3.603695 | 5.44369 | 7.155913 | 6.338363 | 2.869532 | high |
| GSM1527135 | 2.058333333 | 1 | 5.4112 | 3.939093 | 4.11152 | 3.865653 | 5.25867 | 6.285448 | 5.878509 | 2.502501 | low |
| GSM1527143 | 1.933333333 | 1 | 7.576391 | 4.187132 | 4.273881 | 4.171683 | 4.413213 | 7.040103 | 6.514582 | 2.786634 | high |
| GSM1527210 | 1.908333333 | 1 | 5.722437 | 3.877102 | 4.857492 | 3.336324 | 5.723345 | 9.328588 | 6.5776 | 2.787757 | high |
| GSM1527173 | 1.441666667 | 0 | 6.991436 | 5.389934 | 4.303576 | 5.012739 | 4.240436 | 8.406528 | 5.725856 | 2.821458 | high |
| GSM1527187 | 0.808333333 | 0 | 5.909121 | 6.161254 | 4.235181 | 5.237554 | 5.519255 | 7.676161 | 5.196147 | 2.765758 | high |
| GSM1527234 | 0.266666667 | 1 | 4.533483 | 3.09439 | 3.230438 | 3.558867 | 4.60586 | 6.278443 | 4.280635 | 2.129708 | low |
| GSM1527123 | 1.625 | 1 | 6.311653 | 4.823073 | 3.747708 | 3.822217 | 5.267256 | 6.834334 | 5.76665 | 2.57739 | high |
| GSM1527127 | 1.333333333 | 1 | 5.114149 | 3.151209 | 3.767528 | 3.231771 | 5.047003 | 7.321574 | 5.746641 | 2.386066 | low |
| GSM1527131 | 0.233333333 | 1 | 6.325702 | 4.850513 | 3.587627 | 4.33392 | 4.454011 | 8.123638 | 4.857062 | 2.553879 | low |
