# Supplementary material for: Identifying and Validating an Acidosis-Related Signature Associated with Prognosis and Tumor Immune Infiltration Characteristics in Pancreatic Carcinoma
Source: J Immunol Res. 2021 Dec 28;2021:3821055. doi: 10.1155/2021/3821055 (PMC8727107; doi:10.1155/2021/3821055)
Supplement: Supplementary Materials — Figure S1: the flow chart of the present study. Figure S2: comparisons of ARI risk groups between different clinical subgroups in TCGA-PAAD. Comparisons of the distribution differences of the acidosis-related index (ARI) risk groups among tumor grade (a), Residual_Tumor (b), Tumor_Status (c), and Progressed (d), respectively. (e) Kaplan–Meier curves and log-rank test of disease-free survival (DFS) outcomes between ARI high-risk and low-risk groups. ARI: acidosis-related index; DFS: disease-free survival. Figure S3: comparisons of the expression levels of the seven key genes in the acidosis-related signature. Figure S4: overall survival analyses of the seven key genes in the acidosis-related signature in TCGA-PAAD. Figure S5: significantly enriched pathways of immunologic signature gene sets in the acidosis-related high-risk group in TCGA-PAAD. Figure S6: correlation analyses between the ARI risk scores and TIDE scores. Pearson correlation analyses between the ARI risk scores and TIDE scores in TCGA-PAAD (a) and GSE62452 (b). ARI: acidosis-related index; TIDE: tumor immune dysfunction and exclusion. Table S1: clinicopathological characteristics of patients enrolled in the present study. Table S2: the specific gene signatures of 24 immune cells. Table S3: the results of the LASSO Cox regression. Table S4: acidosis-related risk scores of patients in TCGA-PAAD. Table S5: acidosis-related risk scores of patients in GSE62452. Table S6: TIDE scores of patients in TCGA-PAAD. Table S7: TIDE scores of patients in GSE62452. [file 3821055.f1.zip › Supplemental Table S6.docx]

| **Table S6 TIDE scores of 168 patients in TCGA-PAAD** | | | | | | |
| --- | --- | --- | --- | --- | --- | --- |
| id | riskScore | risk | Responder | TIDE score | Dysfunction | Exclusion |
| TCGA-IB-7652 | 2.1183415 | low | TRUE | -0.88 | -0.43 | -0.88 |
| TCGA-IB-AAUV | 1.3936682 | low | FALSE | 0.43 | 0.43 | 0.78 |
| TCGA-FB-AAQ3 | 2.0867209 | low | FALSE | 0.34 | -0.8 | 0.34 |
| TCGA-IB-7890 | 2.5787992 | high | FALSE | 1.76 | -1.03 | 1.76 |
| TCGA-XD-AAUI | 2.2671994 | high | FALSE | 0 | 0.27 | 0 |
| TCGA-2J-AABK | 1.9345004 | low | TRUE | -0.95 | -0.51 | -0.95 |
| TCGA-IB-A5SS | 2.8137487 | high | FALSE | 1.74 | -0.03 | 1.74 |
| TCGA-FB-AAPZ | 2.4269095 | high | TRUE | -0.13 | -0.13 | -0.47 |
| TCGA-2J-AABI | 2.4788348 | high | FALSE | 0.52 | 0.25 | 0.52 |
| TCGA-HV-A5A6 | 2.1504638 | high | FALSE | 1.03 | -1.08 | 1.03 |
| TCGA-HZ-8001 | 2.2003353 | high | FALSE | 0.69 | 0.69 | 0.87 |
| TCGA-3E-AAAZ | 2.2649819 | high | FALSE | 1.6 | -0.75 | 1.6 |
| TCGA-IB-7885 | 2.3510939 | high | FALSE | 0.12 | 0.12 | 0.69 |
| TCGA-IB-7651 | 2.473971 | high | TRUE | -0.43 | -0.43 | -0.52 |
| TCGA-US-A779 | 2.1156306 | low | TRUE | -0.38 | 0.22 | -0.38 |
| TCGA-HZ-7925 | 2.346671 | high | FALSE | 1.71 | -0.81 | 1.71 |
| TCGA-IB-7886 | 2.7218668 | high | TRUE | -1.18 | -1.18 | 0.35 |
| TCGA-IB-8127 | 2.5786171 | high | TRUE | -0.15 | -0.75 | -0.15 |
| TCGA-HZ-8637 | 1.9414485 | low | FALSE | 0.25 | 0.25 | -2.63 |
| TCGA-HZ-A77P | 1.701704 | low | FALSE | 0.18 | 0.18 | 0.82 |
| TCGA-IB-7646 | 2.7973564 | high | FALSE | 0.44 | -1.21 | 0.44 |
| TCGA-HV-A5A3 | 2.3540507 | high | TRUE | -0.01 | -0.03 | -0.01 |
| TCGA-IB-A6UG | 2.1247477 | low | TRUE | -0.23 | 0.57 | -0.23 |
| TCGA-3A-A9J0 | 2.1143719 | low | FALSE | 0.72 | -0.25 | 0.72 |
| TCGA-IB-AAUQ | 2.3917562 | high | FALSE | 1.35 | 0.09 | 1.35 |
| TCGA-Q3-AA2A | 2.1187361 | low | TRUE | -0.27 | 0.1 | -0.27 |
| TCGA-FB-AAQ2 | 2.7244426 | high | FALSE | 1.21 | -1.31 | 1.21 |
| TCGA-2L-AAQM | 0.9571532 | low | TRUE | -0.22 | 0.31 | -0.22 |
| TCGA-IB-7649 | 1.8502524 | low | FALSE | 0.03 | 0.03 | -0.76 |
| TCGA-HZ-8317 | 1.9134137 | low | FALSE | 0.41 | -0.5 | 0.41 |
| TCGA-3A-A9I9 | 1.7611874 | low | TRUE | -1.02 | 1.55 | -1.02 |
| TCGA-FB-AAPY | 1.9209392 | low | TRUE | -1.61 | 2.09 | -1.61 |
| TCGA-H6-8124 | 2.3339524 | high | FALSE | 2.17 | -1.55 | 2.17 |
| TCGA-3E-AAAY | 1.8924317 | low | FALSE | 0.69 | 0.69 | -0.95 |
| TCGA-HZ-A9TJ | 1.7060521 | low | TRUE | -0.22 | -0.81 | -0.22 |
| TCGA-LB-A8F3 | 1.4157867 | low | FALSE | 0.64 | -0.13 | 0.64 |
| TCGA-US-A77E | 2.2644115 | high | FALSE | 0.84 | -0.26 | 0.84 |
| TCGA-YY-A8LH | 2.393869 | high | TRUE | -0.38 | -0.32 | -0.38 |
| TCGA-IB-7654 | 1.8288761 | low | FALSE | 0.13 | -0.23 | 0.13 |
| TCGA-FB-A78T | 2.1441567 | high | TRUE | -1.65 | 0.13 | -1.65 |
| TCGA-2J-AAB8 | 2.1715052 | high | FALSE | 1.46 | -1.16 | 1.46 |
| TCGA-FB-A4P6 | 1.8835672 | low | TRUE | -0.91 | 0.15 | -0.91 |
| TCGA-FB-A7DR | 2.3538155 | high | FALSE | 1.19 | 0.14 | 1.19 |
| TCGA-2L-AAQL | 1.8383251 | low | FALSE | 0.03 | 1.1 | 0.03 |
| TCGA-HZ-7920 | 1.632111 | low | TRUE | -0.51 | -0.51 | -0.19 |
| TCGA-FB-AAPU | 2.4051187 | high | TRUE | -0.44 | -0.93 | -0.44 |
| TCGA-IB-A6UF | 2.2958784 | high | FALSE | 0.83 | -1.24 | 0.83 |
| TCGA-OE-A75W | 2.2567178 | high | FALSE | 1.05 | 1.05 | 0.32 |
| TCGA-3A-A9IZ | 2.773588 | high | FALSE | 1.06 | -1.34 | 1.06 |
| TCGA-2J-AABU | 2.7167156 | high | TRUE | -0.22 | -0.22 | 1.61 |
| TCGA-HZ-7924 | 2.1372196 | high | TRUE | -0.97 | -0.97 | -0.88 |
| TCGA-HZ-7289 | 2.2219959 | high | TRUE | -0.13 | -0.48 | -0.13 |
| TCGA-HZ-7926 | 2.3955928 | high | TRUE | -1.07 | -1.07 | -0.5 |
| TCGA-RB-AA9M | 2.2270144 | high | FALSE | 0.9 | -1.19 | 0.9 |
| TCGA-2J-AABH | 1.7843527 | low | TRUE | -0.64 | 0.56 | -0.64 |
| TCGA-IB-7645 | 2.0624563 | low | TRUE | -0.2 | -0.2 | -0.71 |
| TCGA-2J-AABA | 2.3256449 | high | FALSE | 1.09 | -0.35 | 1.09 |
| TCGA-HZ-A4BH | 2.3536035 | high | FALSE | 0.08 | 0.08 | -0.18 |
| TCGA-FB-AAPQ | 2.1105238 | low | TRUE | -0.42 | -0.81 | -0.42 |
| TCGA-FB-AAQ0 | 2.2129487 | high | FALSE | 0.24 | -0.57 | 0.24 |
| TCGA-2J-AAB1 | 2.5375319 | high | FALSE | 0.97 | 0.97 | -0.76 |
| TCGA-H6-A45N | 2.020729 | low | FALSE | 1 | 1 | -0.54 |
| TCGA-F2-A44H | 1.2987452 | low | TRUE | -0.04 | 0.98 | -0.04 |
| TCGA-IB-7644 | 2.5367061 | high | FALSE | 0.55 | -1.2 | 0.55 |
| TCGA-3A-A9IB | 2.4830851 | high | FALSE | 1.72 | -0.43 | 1.72 |
| TCGA-IB-AAUW | 1.8371387 | low | TRUE | -0.1 | -0.1 | -1.02 |
| TCGA-HZ-7919 | 2.564537 | high | FALSE | 0.34 | -1.15 | 0.34 |
| TCGA-FB-AAPS | 1.71319 | low | FALSE | 1.16 | 1.16 | 1.13 |
| TCGA-LB-A7SX | 2.1060858 | low | TRUE | -0.01 | -0.09 | -0.01 |
| TCGA-IB-AAUS | 1.8680782 | low | FALSE | 1.31 | 1.31 | -0.99 |
| TCGA-HV-A7OP | 1.0988021 | low | TRUE | -0.28 | 0.08 | -0.28 |
| TCGA-FB-AAQ1 | 2.3591743 | high | FALSE | 0.02 | -0.22 | 0.02 |
| TCGA-IB-7888 | 1.9116418 | low | FALSE | 0.01 | 0.01 | -0.09 |
| TCGA-F2-7273 | 1.832418 | low | FALSE | 0.1 | 0.1 | -0.61 |
| TCGA-2L-AAQI | 2.6233956 | high | FALSE | 0.06 | -0.91 | 0.06 |
| TCGA-XD-AAUG | 1.4176806 | low | FALSE | 1.65 | 0.47 | 1.65 |
| TCGA-3A-A9IS | 0.982088 | low | TRUE | -0.37 | -0.56 | -0.37 |
| TCGA-IB-AAUT | 1.5819771 | low | FALSE | 1.14 | 1.14 | -1.37 |
| TCGA-Z5-AAPL | 1.8818985 | low | FALSE | 0.9 | 0.9 | -2.29 |
| TCGA-HZ-A4BK | 2.1998416 | high | FALSE | 0.86 | 0.86 | -1.08 |
| TCGA-US-A776 | 2.0987519 | low | TRUE | -0.43 | -0.87 | -0.43 |
| TCGA-IB-7889 | 2.0322524 | low | FALSE | 0.07 | 0.07 | -0.49 |
| TCGA-IB-AAUR | 1.7748551 | low | FALSE | 1.86 | 1.86 | -1.48 |
| TCGA-IB-A7M4 | 2.6726708 | high | FALSE | 0.32 | -0.57 | 0.32 |
| TCGA-IB-A5ST | 1.7317335 | low | FALSE | 0.92 | 0.92 | -0.68 |
| TCGA-3A-A9IU | 2.5960454 | high | FALSE | 0.38 | -0.49 | 0.38 |
| TCGA-3A-A9IJ | 0.5825794 | low | FALSE | 0.12 | 0.28 | 0.12 |
| TCGA-3A-A9IH | 2.4069639 | high | FALSE | 0.51 | -0.25 | 0.51 |
| TCGA-F2-A8YN | 2.1783708 | high | FALSE | 0.52 | -0.46 | 0.52 |
| TCGA-HZ-A49I | 2.2435085 | high | TRUE | -0.81 | 0.87 | -0.81 |
| TCGA-HV-A5A4 | 2.1653956 | high | FALSE | 0.31 | 0.1 | 0.31 |
| TCGA-HZ-8636 | 2.3131781 | high | FALSE | 0.28 | -1.09 | 0.28 |
| TCGA-2J-AABO | 2.3079745 | high | FALSE | 1.83 | -0.19 | 1.83 |
| TCGA-FB-A5VM | 2.7096549 | high | FALSE | 0.64 | 0.64 | 0.82 |
| TCGA-HZ-8315 | 2.3006872 | high | TRUE | -0.54 | -0.54 | -0.39 |
| TCGA-F2-6880 | 0.5425152 | low | TRUE | -1.09 | 1.26 | -1.09 |
| TCGA-2L-AAQJ | 2.1937077 | high | TRUE | -0.68 | 0.48 | -0.68 |
| TCGA-HZ-8005 | 2.6120898 | high | FALSE | 1.53 | -0.77 | 1.53 |
| TCGA-2J-AAB9 | 2.1397928 | high | FALSE | 0.89 | 0.63 | 0.89 |
| TCGA-XN-A8T5 | 1.7076994 | low | FALSE | 1.22 | 1.22 | 0.23 |
| TCGA-HZ-8638 | 2.1802386 | high | TRUE | -1.4 | -1.4 | -0.62 |
| TCGA-L1-A7W4 | 3.0299362 | high | FALSE | 1.24 | -1.62 | 1.24 |
| TCGA-3A-A9IX | 1.7513543 | low | TRUE | -0.64 | 0.74 | -0.64 |
| TCGA-XD-AAUH | 1.6599485 | low | FALSE | 0.96 | 0.96 | -0.58 |
| TCGA-HZ-A77O | 2.203745 | high | FALSE | 0.92 | -0.01 | 0.92 |
| TCGA-IB-A5SO | 2.0886803 | low | TRUE | -0.18 | 1.69 | -0.18 |
| TCGA-F2-6879 | 2.5677941 | high | FALSE | 0.62 | -1.41 | 0.62 |
| TCGA-2J-AAB4 | 2.3649372 | high | TRUE | -0.83 | 0.2 | -0.83 |
| TCGA-US-A774 | 2.1342724 | low | FALSE | 0.76 | 0.76 | 0.31 |
| TCGA-IB-8126 | 1.1326434 | low | FALSE | 1.09 | 1.09 | -1.34 |
| TCGA-F2-A44G | 2.4488604 | high | FALSE | 0.2 | -0.74 | 0.2 |
| TCGA-2J-AABR | 2.1216863 | low | FALSE | 0.63 | 0.63 | -0.79 |
| TCGA-IB-A5SP | 2.0489747 | low | TRUE | -1.02 | 0.54 | -1.02 |
| TCGA-H8-A6C1 | 2.051646 | low | TRUE | -0.53 | 0.03 | -0.53 |
| TCGA-2J-AABE | 1.7907423 | low | FALSE | 0.27 | 0.42 | 0.27 |
| TCGA-RB-A7B8 | 1.9701569 | low | FALSE | 0.63 | 0.6 | 0.63 |
| TCGA-IB-AAUO | 2.6555839 | high | TRUE | -0.12 | 0.31 | -0.12 |
| TCGA-2J-AAB6 | 2.7499452 | high | FALSE | 1.48 | -0.42 | 1.48 |
| TCGA-2J-AABF | 2.422966 | high | FALSE | 0.29 | 0.29 | -1.86 |
| TCGA-HZ-8002 | 2.0779007 | low | TRUE | -1.15 | -1.15 | 0.22 |
| TCGA-FB-AAQ6 | 2.003701 | low | FALSE | 0.55 | -1 | 0.55 |
| TCGA-IB-7891 | 1.9503001 | low | TRUE | -0.03 | -0.03 | -0.69 |
| TCGA-YB-A89D | 2.1305003 | low | FALSE | 0.77 | 0.62 | 0.77 |
| TCGA-IB-A5SQ | 2.5420569 | high | FALSE | 1.47 | 0.4 | 1.47 |
| TCGA-2J-AABP | 1.8652179 | low | FALSE | 0.01 | 0.01 | 1 |
| TCGA-IB-A7LX | 2.7366327 | high | FALSE | 0.53 | -0.98 | 0.53 |
| TCGA-US-A77J | 1.6103739 | low | FALSE | 2 | 2 | -0.68 |
| TCGA-HV-AA8X | 1.9254789 | low | FALSE | 0.2 | -0.85 | 0.2 |
| TCGA-HZ-A77Q | 1.8978598 | low | FALSE | 0.99 | 0.99 | 0.24 |
| TCGA-2J-AABV | 0.8407052 | low | TRUE | -0.66 | 1.55 | -0.66 |
| TCGA-LB-A9Q5 | 1.55964 | low | TRUE | -1.09 | 1.58 | -1.09 |
| TCGA-S4-A8RO | 2.4206262 | high | FALSE | 0.3 | -1.27 | 0.3 |
| TCGA-3A-A9IC | 2.1512677 | high | FALSE | 1.95 | 0.22 | 1.95 |
| TCGA-HZ-8519 | 1.60815 | low | TRUE | -0.27 | -0.24 | -0.27 |
| TCGA-3A-A9IR | 0.7256365 | low | TRUE | -0.52 | -0.31 | -0.52 |
| TCGA-HV-AA8V | 2.0414948 | low | FALSE | 1.35 | 1 | 1.35 |
| TCGA-Q3-A5QY | 1.560831 | low | FALSE | 2.15 | 2.15 | -2.09 |
| TCGA-3A-A9IL | 0.7592699 | low | TRUE | -0.27 | 0.64 | -0.27 |
| TCGA-HZ-A49G | 2.0436612 | low | TRUE | -1.68 | 0.92 | -1.68 |
| TCGA-S4-A8RP | 2.2215213 | high | TRUE | -0.3 | -0.12 | -0.3 |
| TCGA-HZ-A49H | 1.5993841 | low | FALSE | 0.84 | 0.84 | -0.41 |
| TCGA-S4-A8RM | 2.3432856 | high | TRUE | -1.05 | -0.81 | -1.05 |
| TCGA-IB-AAUP | 2.0884207 | low | FALSE | 1.33 | 1.33 | -0.94 |
| TCGA-IB-AAUN | 2.4688209 | high | FALSE | 0.7 | -0.41 | 0.7 |
| TCGA-HZ-7918 | 1.9480994 | low | TRUE | -0.28 | -0.28 | -1.2 |
| TCGA-IB-7887 | 2.3487492 | high | TRUE | -1.08 | -1.08 | 1.06 |
| TCGA-3A-A9IN | 0.7881667 | low | TRUE | -0.16 | 0.97 | -0.16 |
| TCGA-F2-7276 | 1.963173 | low | TRUE | -0.29 | -0.41 | -0.29 |
| TCGA-HV-A5A5 | 1.9298306 | low | TRUE | -0.87 | 0.91 | -0.87 |
| TCGA-IB-7893 | 2.7880772 | high | FALSE | 2.32 | -1.68 | 2.32 |
| TCGA-2L-AAQE | 2.5065067 | high | FALSE | 0.67 | 0.17 | 0.67 |
| TCGA-HZ-8003 | 1.5542244 | low | TRUE | -1.62 | 1.24 | -1.62 |
| TCGA-M8-A5N4 | 2.3628159 | high | TRUE | -0.56 | 0.59 | -0.56 |
| TCGA-2J-AABT | 1.9707736 | low | FALSE | 0.05 | 0.58 | 0.05 |
| TCGA-IB-AAUU | 2.4495966 | high | TRUE | -0.9 | -0.08 | -0.9 |
| TCGA-FB-A545 | 2.7722233 | high | FALSE | 1.56 | -0.75 | 1.56 |
| TCGA-PZ-A5RE | 2.3048261 | high | TRUE | -0.31 | 1.14 | -0.31 |
| TCGA-HV-A7OL | 2.1535514 | high | TRUE | -0.64 | 1.34 | -0.64 |
| TCGA-IB-7897 | 1.9005855 | low | TRUE | -0.39 | -0.39 | -0.55 |
| TCGA-XN-A8T3 | 2.2845032 | high | FALSE | 1.41 | -0.38 | 1.41 |
| TCGA-HZ-7923 | 1.648633 | low | FALSE | 0.74 | 0.74 | -1.19 |
| TCGA-FB-A4P5 | 2.0853398 | low | FALSE | 1.72 | 1.72 | -0.86 |
| TCGA-2L-AAQA | 2.4875888 | high | FALSE | 0.52 | -1.05 | 0.52 |
| TCGA-3A-A9IV | 1.2459913 | low | FALSE | 0.22 | -0.27 | 0.22 |
| TCGA-3A-A9I7 | 2.0374995 | low | TRUE | -0.05 | 0.9 | -0.05 |
| TCGA-FB-AAPP | 2.6078578 | high | TRUE | -0.47 | -1.1 | -0.47 |
| TCGA-F2-A7TX | 2.6893567 | high | TRUE | -0.21 | -0.21 | -0.53 |
| TCGA-XD-AAUL | 2.451993 | high | FALSE | 0.56 | 0.38 | 0.56 |
