# Supplementary material for: Identifying and Validating an Acidosis-Related Signature Associated with Prognosis and Tumor Immune Infiltration Characteristics in Pancreatic Carcinoma
Source: J Immunol Res. 2021 Dec 28;2021:3821055. doi: 10.1155/2021/3821055 (PMC8727107; doi:10.1155/2021/3821055)
Supplement: Supplementary Materials — Figure S1: the flow chart of the present study. Figure S2: comparisons of ARI risk groups between different clinical subgroups in TCGA-PAAD. Comparisons of the distribution differences of the acidosis-related index (ARI) risk groups among tumor grade (a), Residual_Tumor (b), Tumor_Status (c), and Progressed (d), respectively. (e) Kaplan–Meier curves and log-rank test of disease-free survival (DFS) outcomes between ARI high-risk and low-risk groups. ARI: acidosis-related index; DFS: disease-free survival. Figure S3: comparisons of the expression levels of the seven key genes in the acidosis-related signature. Figure S4: overall survival analyses of the seven key genes in the acidosis-related signature in TCGA-PAAD. Figure S5: significantly enriched pathways of immunologic signature gene sets in the acidosis-related high-risk group in TCGA-PAAD. Figure S6: correlation analyses between the ARI risk scores and TIDE scores. Pearson correlation analyses between the ARI risk scores and TIDE scores in TCGA-PAAD (a) and GSE62452 (b). ARI: acidosis-related index; TIDE: tumor immune dysfunction and exclusion. Table S1: clinicopathological characteristics of patients enrolled in the present study. Table S2: the specific gene signatures of 24 immune cells. Table S3: the results of the LASSO Cox regression. Table S4: acidosis-related risk scores of patients in TCGA-PAAD. Table S5: acidosis-related risk scores of patients in GSE62452. Table S6: TIDE scores of patients in TCGA-PAAD. Table S7: TIDE scores of patients in GSE62452. [file 3821055.f1.zip › Supplemental Table S7.docx]

| **Table S7 TIDE scores of 63 patients in GSE62452** | | | | | | |
| --- | --- | --- | --- | --- | --- | --- |
| id | risk score | risk | Responder | TIDE | Dysfunction | Exclusion |
| GSM1527183 | 2.817126 | high | FALSE | 1.53 | 0.18 | 1.53 |
| GSM1527196 | 2.659418 | high | TRUE | -0.82 | 1.46 | -0.82 |
| GSM1527207 | 2.722501 | high | FALSE | 0.42 | -0.46 | 0.42 |
| GSM1527232 | 2.198086 | low | TRUE | -0.68 | 0.26 | -0.68 |
| GSM1527105 | 2.355378 | low | FALSE | 0.05 | 0.65 | 0.05 |
| GSM1527109 | 2.814371 | high | FALSE | 0.89 | 0.08 | 0.89 |
| GSM1527137 | 1.984719 | low | TRUE | -0.62 | -0.26 | -0.62 |
| GSM1527141 | 2.548125 | low | FALSE | 0.81 | 0.99 | 0.81 |
| GSM1527151 | 2.369757 | low | FALSE | 0.2 | -0.4 | 0.2 |
| GSM1527167 | 2.629421 | high | FALSE | 2.37 | -0.2 | 2.37 |
| GSM1527209 | 2.771109 | high | TRUE | -0.17 | -1.56 | -0.17 |
| GSM1527212 | 2.25615 | low | FALSE | 0.9 | 0.9 | 1.36 |
| GSM1527218 | 3.079502 | high | FALSE | 0.52 | -1.17 | 0.52 |
| GSM1527230 | 2.172685 | low | FALSE | 0.57 | -0.42 | 0.57 |
| GSM1527139 | 1.879217 | low | TRUE | -0.54 | -0.58 | -0.54 |
| GSM1527145 | 2.517012 | low | TRUE | -0.87 | 0.1 | -0.87 |
| GSM1527147 | 2.175493 | low | TRUE | -0.23 | 0.99 | -0.23 |
| GSM1527149 | 2.289326 | low | TRUE | -1.03 | -0.25 | -1.03 |
| GSM1527155 | 2.665125 | high | FALSE | 1.09 | 1.09 | -1.07 |
| GSM1527157 | 2.485644 | low | FALSE | 0.01 | 0.35 | 0.01 |
| GSM1527159 | 2.611163 | high | TRUE | -0.17 | -0.17 | 0.17 |
| GSM1527161 | 2.396862 | low | TRUE | -1.07 | 1.92 | -1.07 |
| GSM1527163 | 2.736986 | high | FALSE | 0.55 | -0.11 | 0.55 |
| GSM1527165 | 2.61428 | high | FALSE | 0.36 | 0.78 | 0.36 |
| GSM1527169 | 2.753774 | high | TRUE | -0.06 | -0.24 | -0.06 |
| GSM1527171 | 2.619459 | high | FALSE | 1.98 | 1.98 | -2.17 |
| GSM1527175 | 2.672981 | high | TRUE | -1.14 | 1.45 | -1.14 |
| GSM1527177 | 2.580017 | high | FALSE | 1.14 | -1.49 | 1.14 |
| GSM1527179 | 2.465827 | low | TRUE | -0.24 | -1.08 | -0.24 |
| GSM1527181 | 2.703083 | high | TRUE | -0.71 | 0.2 | -0.71 |
| GSM1527185 | 1.967678 | low | TRUE | -0.6 | 0.31 | -0.6 |
| GSM1527189 | 2.596665 | high | FALSE | 0.75 | -1.17 | 0.75 |
| GSM1527191 | 2.145689 | low | FALSE | 1.64 | 1.64 | 0.56 |
| GSM1527193 | 2.376873 | low | TRUE | -0.07 | -0.03 | -0.07 |
| GSM1527198 | 2.776236 | high | FALSE | 0.92 | -0.74 | 0.92 |
| GSM1527200 | 2.060232 | low | FALSE | 0.6 | 0.6 | -1.48 |
| GSM1527202 | 2.608801 | high | FALSE | 1.09 | 0.08 | 1.09 |
| GSM1527204 | 2.819801 | high | FALSE | 1.35 | -0.45 | 1.35 |
| GSM1527205 | 2.259139 | low | TRUE | -0.89 | -0.58 | -0.89 |
| GSM1527213 | 2.655598 | high | TRUE | -0.8 | -0.49 | -0.8 |
| GSM1527215 | 2.19447 | low | TRUE | -0.37 | 0.37 | -0.37 |
| GSM1527216 | 2.251701 | low | TRUE | -0.22 | -0.89 | -0.22 |
| GSM1527219 | 2.533329 | low | FALSE | 0.14 | 0.14 | -0.33 |
| GSM1527220 | 2.796734 | high | FALSE | 0.66 | -1.65 | 0.66 |
| GSM1527223 | 2.260059 | low | FALSE | 0.02 | -0.61 | 0.02 |
| GSM1527225 | 2.4648 | low | FALSE | 1.16 | -0.24 | 1.16 |
| GSM1527227 | 2.136886 | low | TRUE | -0.25 | 0.54 | -0.25 |
| GSM1527107 | 3.003939 | high | FALSE | 0.73 | -0.59 | 0.73 |
| GSM1527111 | 2.041009 | low | TRUE | -0.2 | 1.15 | -0.2 |
| GSM1527115 | 2.639572 | high | FALSE | 0.87 | -0.98 | 0.87 |
| GSM1527117 | 2.587112 | high | FALSE | 2.52 | 0.65 | 2.52 |
| GSM1527125 | 2.788535 | high | TRUE | -0.53 | -0.42 | -0.53 |
| GSM1527129 | 2.55219 | low | TRUE | 0 | 0.44 | 0 |
| GSM1527133 | 2.869532 | high | TRUE | -0.56 | -1.64 | -0.56 |
| GSM1527135 | 2.502501 | low | FALSE | 1.14 | 1.14 | -0.49 |
| GSM1527143 | 2.786634 | high | FALSE | 0.19 | 0.19 | -0.99 |
| GSM1527210 | 2.787757 | high | TRUE | -0.56 | -1.12 | -0.56 |
| GSM1527173 | 2.821458 | high | FALSE | 1.74 | -1.41 | 1.74 |
| GSM1527187 | 2.765758 | high | TRUE | -0.07 | -0.23 | -0.07 |
| GSM1527234 | 2.129708 | low | FALSE | 0.12 | 1.07 | 0.12 |
| GSM1527123 | 2.57739 | high | TRUE | -0.75 | -0.46 | -0.75 |
| GSM1527127 | 2.386066 | low | TRUE | -1.38 | -0.76 | -1.38 |
| GSM1527131 | 2.553879 | low | FALSE | 0.96 | 0.41 | 0.96 |
